# Older adults across the globe exhibit increased prosocial behavior but also greater in-group preferences

**DOI:** 10.1038/s43587-021-00118-3

**Published:** 2021-10-11

**Authors:** Jo Cutler, Jonas P. Nitschke, Claus Lamm, Patricia L. Lockwood

**Affiliations:** 1 Centre for Human Brain Health, University of Birmingham, Birmingham, UK; 2 Department of Experimental Psychology, University of Oxford, Oxford, UK; 3 Wellcome Centre for Integrative Neuroimaging, Department of Experimental Psychology, University of Oxford, Oxford, UK; 4 Department of Cognition, Emotion, and Methods in Psychology, University of Vienna, Vienna, Austria; 5 Department of Psychology, McGill University, Montreal, Quebec Canada; 6 Christ Church, University of Oxford, Oxford, UK

**Keywords:** Psychology, Social sciences, Ageing

## Abstract

Population aging is a global phenomenon with substantial implications across society^1,2^. Prosocial behaviors—actions that benefit others—promote mental and physical health across the lifespan^3,4^ and can save lives during the COVID-19 pandemic. We examined whether age predicts prosociality in a preregistered global study (46,576 people aged 18–99 across 67 countries) using two acutely relevant measures: distancing during COVID-19 and willingness to donate to hypothetical charities. Age positively predicted prosociality on both measures, with increased distancing and donations among older adults. However, older adults were more in-group focused than younger adults in choosing who to help, making larger donations to national over international charities and reporting increased in-group preferences. In-group preferences helped explain greater national over international donations. Results were robust to several control analyses and internal replication. Our findings have vital implications for predicting the social and economic impacts of aging populations, increasing compliance with public health measures and encouraging charitable donations.

## Abstract (web summary)

Cutler et al. found that age predicted individuals’ likelihood to engage in prosocial behaviors such as social distancing during COVID-19 and charitable donations. Older adults across the world were more willing to help but also demonstrated stronger in-group preferences, donating less to international charities.

## Main

Prosocial behaviors have critical individual and societal impacts^5^. Emerging evidence suggests that older adults might be more prosocial than younger adults^6^ on measures including economic games^7–9^, learning about rewards for others^10^, effortful actions^11^ and charitable donations^12–15^. In line with this, theoretical accounts of lifespan development, such as socioemotional selectivity theory^16^, propose that motivation for socially and emotionally meaningful behaviors increases as a result of age-related differences in goals and priorities^17,18^. However, most research has tested participants from western, educated, industrialized, rich and democratic populations^19^. It is unknown whether increased prosociality is shown by older adults across the world. Moreover, although some studies point to increased prosocial behavior, others find no association^20^ or even heightened negative behaviors, including greater bias toward one’s own emotions^21^, increased stereotyping of outgroups^22^ and less support for foreign aid^23^. Together these findings suggest that age might be associated with both increased positive helping behaviors but also heightened self-serving and in-group preferences.

The coronavirus disease 2019 (COVID-19) global pandemic is a recent and striking demonstration of the link between our actions and life or death consequences for others. While access to a vaccine is limited globally, the primary defenses are behavioral, requiring changes from normal behavior and sacrifices to convenience to reduce social contact^24^. Even with the vaccine, it is essential that people get inoculated. Therefore, governments around the world have stressed the importance of individual behaviors, particularly distancing, for protecting others and largely rely on voluntary adherence.

Here we measured two core aspects of prosociality, framed in the context of the pandemic to ensure the same cause was relevant in all countries and evaluate behaviors that were not established habits. The first measure was self-reported levels of distancing, averaging four items asking how much participants were limiting contact with others^25^. While novel, this measure had good construct validity and strong internal consistency^25^. The second measure assessed another core aspect of prosocial behavior—willingness to donate to charity. We applied the widely used and well-validated dictator game^9,26^ to hypothetical donations^27–29^. Participants stated the percentage of a specified amount of hypothetical money they would: keep; donate to a national charity; and donate to an international charity. The amount was the median daily wage in the participant’s country and charities were described as providing medical support for COVID-19 either “in your own country” or “all over the world.” Critically, manipulating the donation recipient allowed us to assess in-country preference—the difference between donations to the national and the international charity. Data were collected in April and May 2020 (Supplementary Figs. 1 and 2).

To understand any age-related differences in prosocial behavior, socioemotional differences must be separated from non-social factors that also differ with age. If older people distance more, it could be because they have a higher fatality risk from COVID-19 (ref. ^30^). We therefore controlled for participants’ perceived risk of catching the virus, COVID-19 severity at the time of testing and participants’ self-reported physical health. Older adults also have greater accumulated wealth^31^, which could explain any increases in financial prosocial behaviors. Therefore, we controlled for subjective and, where possible, objective wealth. Finally, in addition to differences in prosocial behavior, research suggests that socioemotional traits differ across the lifespan^32^. A range of traits are associated with prosocial behavior^33^, but research on this association across the lifespan is limited. We examined age-related differences in socioemotional traits using established questionnaires. We predicted that older adults would score higher on traits such as optimism, well-being and moral preferences that predict increased prosocial behavior^14^. From findings that in-group preference increases with age^22,23^, we predicted that scores on national identity and collective narcissism (belief in the superiority of one’s country) would be higher in older adults and predict an in-country preference in giving.

We tested these preregistered hypotheses of age-related differences in prosocial behavior and traits using data from 46,576 participants collected as representative samples in 67 countries (see Methods). As preregistered, we randomly created two equal subsamples to enable internal replication^34^. All main results were robust to replication and descriptions apply to both subsample 1 (S1) and subsample 2 (S2) unless otherwise specified. Statistics are provided in the format S1 | S2. All key results also remained when age was adjusted for life expectancy in each country. We confirmed that both prosocial measures had good test–retest reliability one month later in a subset of participants (intraclass correlation for distancing = 0.70, total donations = 0.80; *n* = 448; see Supplementary Methods).

**Fig. 1:**
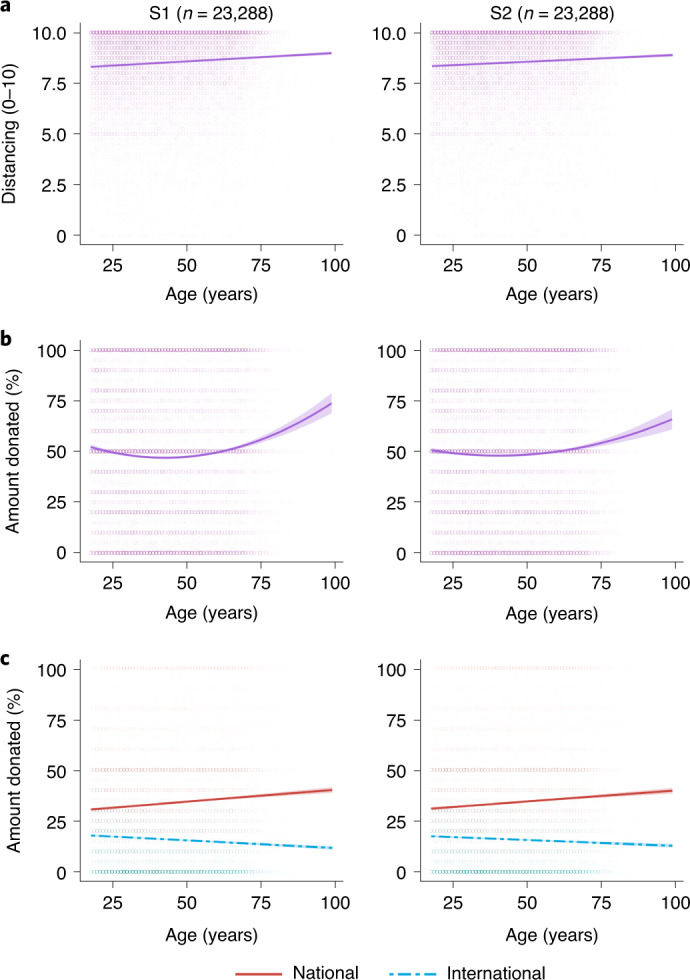
Age predicts greater distancing and charitable donations, but increased preference for national over international charities. **a**,**b**, In both subsample 1 (S1, left) and subsample 2 (S2, right), older age predicted higher rates of distancing (**a**) and hypothetical charitable donations (**b**) when summed across both charities. The relationship between age and total donations is quadratic. **c**, When taking into account charity location, age was positively associated with national donations but negatively associated with international donations. Lines show fitted linear models; shaded areas show 95% CIs; circles show individual data points. Source data

Age positively predicted both distancing and donations in linear mixed-effects models (LMMs; Supplementary Tables 1–3). Older age was associated with increased distancing (Fig. 1a; *β* = 0.10 | 0.10, *P* values < 0.001) and donations (Fig. 1b; *β* = 0.04 | 0.05, *P* values < 0.001). For every increase of 16 years in age, distancing increased by 0.18 | 0.17 on the 11-point scale and donations increased by 1.50% | 1.71%. Women were more prosocial than men on both measures (Supplementary Table 1). Perceived risk was not significantly associated with distancing (*P* = 0.07 | 0.10). An additional, exploratory control model (not preregistered), accounting for participants’ self-reported physical health, replicated all results, and better health predicted greater distancing (Supplementary Table 4). For donations, strikingly, subjective wealth had a negative effect: those who perceived themselves as wealthier donated less (*β* = −0.08 | −0.08, *P* values < 0.001). We also observed significant positive correlations between distancing and donations, consistent with a shared prosocial disposition (Pearson’s *r* = 0.14 | 0.14, *P* values < 0.001).

Next we examined nonlinear age effects. Previous evidence suggested that giving increases with age to a point then declines^12^. A quadratic age term significantly improved our donation models (*χ*^2^(1): 71.22 | 32.07, *P* values < 0.001). However, if donations declined at the oldest ages, this term would be negative, whereas it was positive (*β* = 0.06 | 0.04, *P* values < 0.001). Rather than declining in the oldest participants, donations increased slightly in the youngest participants (Fig. 1b).

After showing that older age is associated with increased prosocial behavior overall, we assessed whether age predicted increased donations to both national and international charities. We ran another LMM predicting donations, again controlling for gender and subjective wealth, including a binary predictor of charity location (national = 0, international = 1) and the interaction with age. Overall, participants showed an in-country preference in giving. Donations to international charities (mean [95% confidence interval (CI)]: 15.92% [15.69%, 16.20%] | 15.97% [15.72%, 16.22%]) were less than half the donations to national charities (33.52% [33.13%, 33.86%] | 33.70% [33.32%, 34.07%]; LMM Cohen’s *d* = −0.71 | −0.72, *P* values < 0.001).

Critically, as predicted, charity location significantly interacted with age (*β* = −0.12 | −0.11, *P* values < 0.001; Fig. 1c and Supplementary Table 3). Separate LMMs showed that age positively predicted national donations (*β* = 0.09 | 0.08, *P* values < 0.001), but negatively predicted international donations (*β* = −0.08 | −0.07, *P* values < 0.001). An increase of 16 years in age corresponded to 2.67% | 2.44% larger national donations but 1.64% | 1.32% smaller international donations. Therefore, older adults showed greater in-country preferences in giving. In a subset of participants (*n* = 2,624, 5 countries), an additional analysis controlling for objective wealth (monthly income) also showed that age positively predicted donations (*β* = 0.11, *P* = 0.02) and in-country preferences (interaction *β* = −0.22, *P* < 0.001; Supplementary Table 5 and Supplementary Fig. 4). As the direction of the age effect was different for national and international donations, we treated these as separate outcomes in the following analyses.

**Fig. 2:**
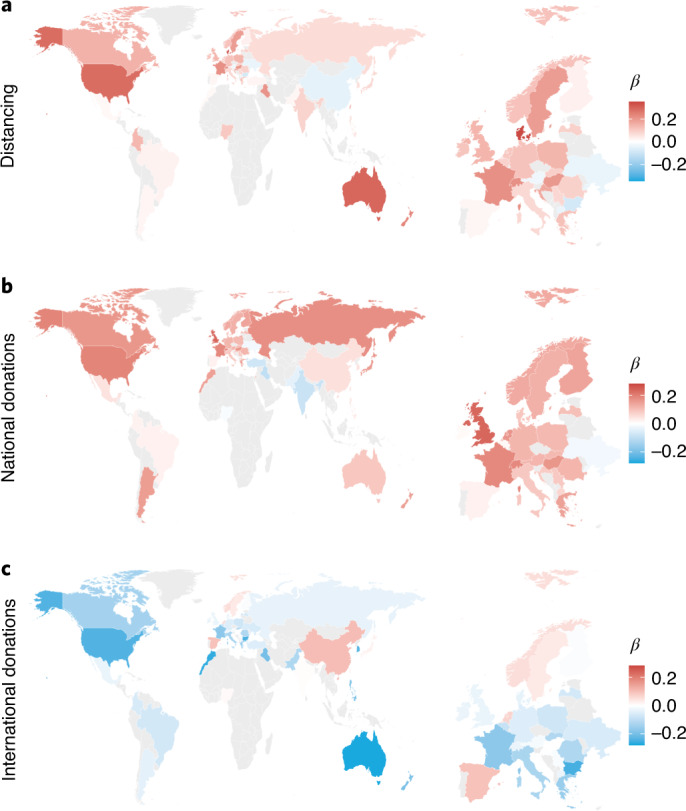
Effects of age on prosocial behavior across the globe. The *β* values are standardized regression coefficients shown on a map of the world (left) and Europe in detail (right). Whether the effect was significant was calculated for the 47 countries with sample sizes above 450 (target of *n* = 500 minus 10% exclusion rate). **a**, Age was positively associated with levels of distancing in most countries and this effect was significant in 29/47 countries (62%). **b**, Older adults made larger hypothetical donations to a national charity in most countries, and this was significant in 27/47 (57%). Interestingly, in 3/47 countries (India, Turkey and Iraq) the relationship between age and national donations was significantly negative. **c**, Age was negatively associated with donations to a hypothetical international charity in many countries, with a significant effect in 23/47 (49%). Again, 3/47 countries (China, Spain and the Netherlands) showed the reverse—age significantly positively predicted international donations. Considering the difference between national and international donations, age significantly predicted this in-country preference in 30/47 (64%) countries. Only in India did older adults show significantly reduced in-country preference. Source data

Our results from analysing all countries together show that age is associated with increased but more in-group-focused prosociality across the world. Next we examined similarities and differences between countries, first with linear models in each country separately. From these we extracted standardized regression coefficients (Fig. 2) and, for the 47 countries with at least 450 participants (500–10% exclusion), the significance for the effect of age. In most countries, across continents and cultures, older age was significantly associated with increased distancing (Fig. 2a) and national donations (Fig. 2b) but increased in-country preferences and lower international donations (Fig. 2c; *P* values < 0.05).

Consistency across countries suggests that age-related differences in prosociality are not driven by country-level confounds. Next we tested whether results remained after controlling for country wealth (gross national income) and COVID-19 severity at the time of data collection. First, we demonstrated no systematic confounds due to timing of data collection and COVID-19 deaths worldwide (Supplementary Figs. 1 and 2). Older adults’ motivation for distancing and donations could be self-centered, as they are more likely to die from the disease. We therefore assessed whether age effects were stronger when the pandemic was more severe. We reasoned that for donations, this would be specific to national donations, so would strengthen the age-related increase in in-country preference. Crucially, the positive effect of age on both distancing (*β* = 0.12 | 0.11, *P* values < 0.001) and donations (*β* = 0.10 | 0.09, *P* values < 0.001) remained significant after controlling for all country-level effects and interactions, as did the interaction between age and charity location (*β* = −0.17 | −0.15, *P* values < 0.001). In the model of distancing, no COVID-19 measures or country wealth significantly moderated the effect of age or significantly predicted distancing (Supplementary Table 6).

For the model of donations, which had multiple three-way interactions, we report the best reduced model (see Methods). Our focus was whether COVID-19 severity moderated the interaction between age and charity location (Supplementary Table 6). If older adults give more nationally because they need medical help with COVID-19, increased in-country preference with age should be strongest where COVID-19 is most severe. In fact, three-way interactions suggest the opposite. Worsening death rates (*β* = 0.05 | 0.06, *P* values < 0.001) and higher death totals (*β* = 0.03 | 0.04, *P* = 0.01 | *<*0.001; note S1 not *P* < 0.01) in the participants’ country were associated with reduced age-related in-country preferences.

**Fig. 3:**
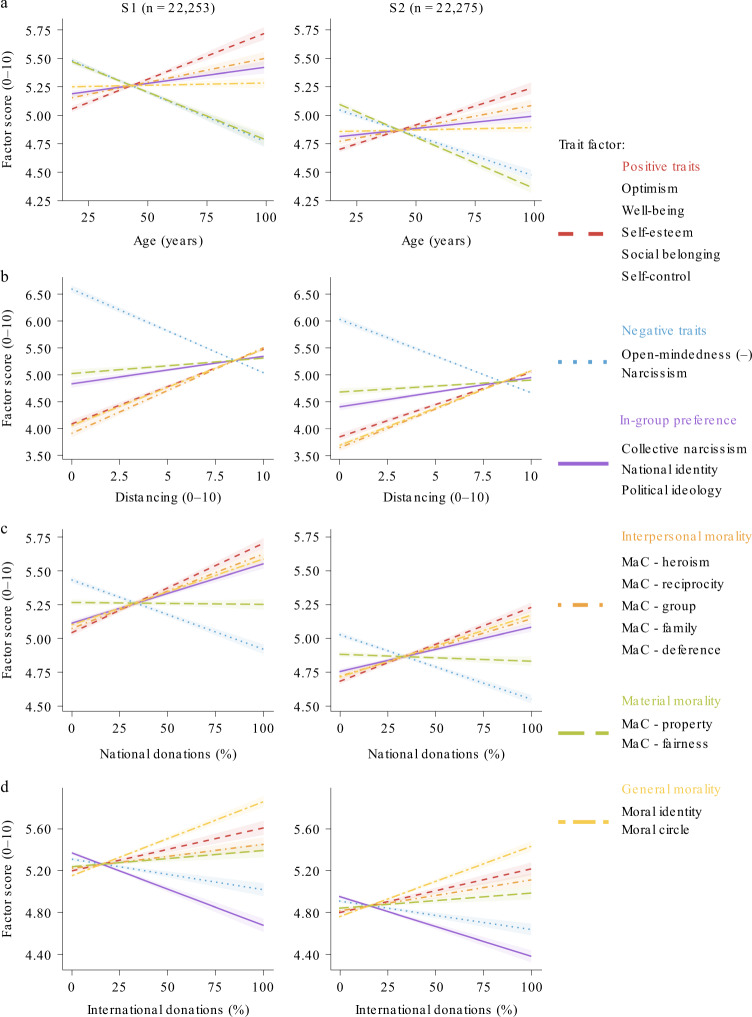
Individual differences in socioemotional traits correlate with age and prosocial behavior. For subsample 1 (S1, left) and subsample 2 (S2, right), we correlated each trait factor with participant age and the three prosocial measures. Trait factors (colored text) were calculated using a factor analysis of 19 traits based on evidence of 6 factors (see Methods). The traits loading onto each factor are listed below their label (see Supplementary Fig. 5 for loadings). MaC, morality-as-cooperation—a scale with seven items that index the importance of different motivations in determining whether something is right or wrong. Political ideology was coded as higher scores representing more right-wing ideology. Higher scores in all other cases represent higher scores on the named trait. **a**, Older age was associated with higher positive traits, interpersonal morality and in-group preference, but lower negative traits and material morality. General morality did not show an association with age. **b**–**d**, The factors also showed correlations with the prosocial measures: distancing (**b**), national donations (**c**) and international donations (**d**). Positive traits, interpersonal morality and general morality each showed a positive relationship with distancing and donations to both charities. Similarly, negative traits were negatively associated with all three prosocial measures. In-group preference was positively associated with distancing and national donations, but negatively associated with international donations (see Supplementary Table 8 for all correlation coefficients and comparisons between prosocial measures in the strength of correlations). Lines show fitted linear models; shaded areas show 95% CIs; sample sizes are the number of participants with data on all 19 trait measures used for the factor analysis. Source data

We have shown age effects on prosocial behavior across countries, which are not accounted for by wealth or self-centered motivations. Next we assessed whether and how individual differences in socioemotional processing over the lifespan relate to prosocial behavior. To test this, we ran a factor analysis on 19 traits. Most showed good test–retest reliability (*n* = 448, Supplementary Methods and Supplementary Table 7). Parallel analysis showed evidence for six factors. On the basis of the measures that loaded onto them (Fig. 3 and Supplementary Fig. 5), we labeled the factors: positive traits, negative traits*,* in-group preference*,* interpersonal morality, material morality and general morality.

**Fig. 4:**
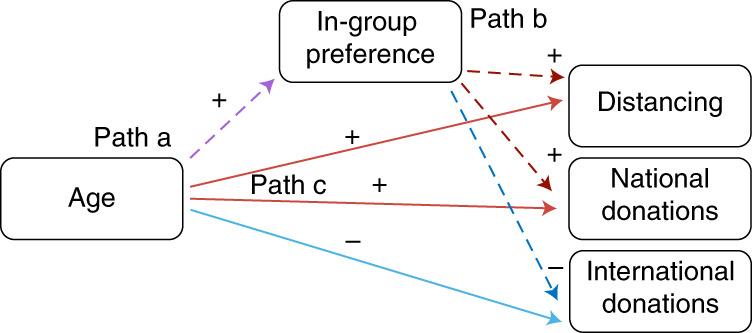
In-group preference partially explains older adults’ larger donations to national charities but smaller donations to international charities. Structural equation models of age effects on each prosocial behavior measure showed significant indirect effects of in-group preference. The models included indirect effects for all six trait factors, as well as the relevant control variable, but here we show a simplified diagram (see Supplementary Fig. 6 for the full model; Supplementary Table 9 for effect sizes—standardized *β* coefficients). Path a: older age predicted higher in-group preference scores (Supplementary Table 9a). Path b: in-group preference was positively related to distancing and national donations but negatively related to international donations (Supplementary Table 9b). Path c: direct effects of age were also positive for distancing and national donations but negative for international donations, as shown in all analyses (Supplementary Table 9c). Dashed lines show paths for indirect effects. Solid lines show direct effects of age on each prosocial behavior. Positive effects on prosocial behaviors are in red; negative effects are in blue. Source data

We established the relevance of these factors through significant correlations with both age (Fig. 3a) and the prosocial measures (Fig. 3b–d and Supplementary Table 8). Older age was associated with higher scores on positive traits, in-group preference and interpersonal morality, but lower scores on negative traits and material morality. Next we used structural equation models to link each prosocial behavior with age, all six trait factors, and the control variables in a single model. Each model (Supplementary Fig. 6) examined possible indirect effects of age on prosocial behavior, via the traits. In other words, do differences in socioemotional traits account for some of the link between age and prosocial behavior? Positive traits, negative traits and in-group preference all showed significant indirect paths from age to prosocial behavior (Supplementary Table 9). Age predicted greater positive traits and in-group preference, but lower negative traits, and these age-related differences accounted for some variance in prosocial behavior. For in-group preference specifically, we were interested in whether this factor differentially predicted donations to the national, compared to the international charity. The indirect effects showed that this was the case. Older adults on average had higher scores on the in-group preference factor and this was positively associated with distancing and national donations, but negatively associated with international donations (Fig. 4).

Understanding the link between age and prosocial behavior is critical for predicting the impacts of an aging society and people’s willingness to comply with public health measures. Here we tested the hypothesis that age predicts both greater prosociality and heightened in-group preferences with data from representative samples across the globe. Older age was associated with greater prosocial behavior on two robust, complementary and acutely relevant measures. However, age was also associated with more in-group focus in who receives help. Older adults donated more to national, but less to international, hypothetical charities than younger adults. The serious risk to older adults from COVID-19 may have prevented in-person prosocial behaviors such as volunteering. However, older adults were particularly willing to help others during a global crisis in terms of compliance with public health measures and support for charities working in their country.

These findings were shown across most countries, replicated across two subsamples, and remained after rigorous control analyses excluding non-social explanations or confounds. Older people could distance more because of their greater risk of serious consequences from COVID-19 (ref. ^30^). We therefore controlled for perceived risk of catching the disease. Lower risk might also result from distancing more. We thus also ran exploratory analyses controlling for self-reported physical health, and in fact, better health positively predicted distancing. Finally, COVID-19 severity in the participants’ country did not moderate the effect of age on distancing. Positive correlations between distancing and both self-reported morality and donations suggest a common other-regarding dimension. While distancing is not exclusively prosocial, complex real-world prosocial behaviors have multiple potential motivations that range in self- or other-focus. Understanding such complexity is vital for research to have applied benefits.

For hypothetical donations, age-related differences could be due to higher wealth among older adults^31^, but controlling for multiple wealth measures did not change the association between age and donations. Hypothetical and non-hypothetical dictator games are comparable in amounts donated and associations with age^8,9^. Both our measures of prosociality are also specific to COVID-19. Collecting data early in the pandemic allowed us to evaluate behaviors newly relevant around the world and measuring donations to COVID-19 charities enabled us to match the national and international causes. It is important that future studies examine prosocial behaviors outside the pandemic for comparison. For instance, our findings could reflect age-related differences in perceived efficacy of distancing and donating during the pandemic or anxiety regarding the economic impact. We believe that our findings transcend the current pandemic and support past empirical and theoretical accounts of increased prosociality among older adults in other contexts^6–16^. Crucially, we demonstrate age-related differences in prosocial behavior in the majority of countries across the globe.

Participants overall gave twice as much to the national as the international charity, and this preference increased with age. Theoretical accounts of older adults’ increased prosociality must therefore explain greater generosity toward charities in the participant’s country, but not abroad. Research suggests that people give more to those perceived as physically close^35^, similar^36^ and in their in-group^37^. Our finding that age predicts in-group and in-country preferences in giving raises interesting links with theories of lifespan development. From the perspective of socioemotional selectivity theory, older people prefer supporting more emotionally meaningful charities^16^, and emotion can bias donation decisions^37^.

Increased in-group and in-country preferences in older age also have crucial practical relevance, particularly as some governments announce foreign aid cuts and need voters’ support. Intriguingly, in-group bias may emerge early in life, with young children displaying preferences for their own gender^38^ and race^39^. Bias can involve both in-group favoritism and outgroup derogation, with the latter including harmful behaviors and biased attitudes^40^. However, children’s in-group preferences have also been linked to sharing and helping^41^. In our data, increased in-group preference helped explain older adults’ larger donations to national charities, not only the difference between giving nationally versus internationally. Future research could assess whether in-group preferences strengthen across the whole lifespan from early childhood to late adulthood, as we observed consistent increases in adulthood across much of the world. More broadly, these results suggest that population aging could affect support for, so likelihood of, reductions in international aid.

Age was also associated with higher levels of positive traits, such as optimism, self-esteem and morality. These findings align with age-related increases in other traits such as empathy^42,43^. Health behaviors such as distancing have also been associated with increased empathy^44^ and prosocial behavior^45^ but decreased ‘dark’ traits such as psychopathy^10,46^. Crucially, examining age, traits and behaviors together showed that age-related differences in traits partially explain differences in prosociality. These findings add to evidence that personality has a role in determining outcomes for individuals and societies^47^. Identifying age-related differences in traits is therefore important for understanding aging. While correlational, our results raise interesting questions about whether cultivating positive traits such as optimism over the lifespan could have wide-reaching social benefits, a hypothesis that future longitudinal studies could test.

If age predicts increased prosocial behavior and socioemotional traits, a further question is whether differences are due to aging, cohort effects or both^48^. In other words, will everyone become more prosocial with age? Longitudinal designs could fully address this question. In the current study, we note three considerations that help to rule out cohort effects. First, age was positively associated with prosocial behavior across the world. Only a handful of countries showed significant reversed effects: age predicting decreased national donations (India, Turkey and Iraq) or increased international donations (China, Spain and the Netherlands). These results do not reveal clear similarities or differences between countries linked to factors such as continent, history or religion. Second, control analyses using age adjusted for life expectancy, in which participants with the same adjusted age were born multiple decades apart, replicated all effects. Third, we specifically focused on newly relevant prosocial behaviors, not established habits that could be more susceptible to cohort effects.

It is important to recognize that our effects were modest but also highly consistent across two subsamples. Given the large sample, we applied stringent significance thresholds and only interpret results that replicated. With the global nature of the pandemic and exponential spread, even marginal changes in preventative behaviors have large impacts. For donations, changes of a few percent with increases of 16 years in age applied to average daily wages are substantial. All 46,576 participants donating 2% more would generate 932 days, 2.5 years, of average wages (£77,782 in the UK) in additional donations.

To conclude, we show that age is a critical determinant of prosocial behavior, notably compliance with public health measures and charitable donations, even in times of global crisis. Accounts of healthy aging should consider this potential increase in beneficial social behavior alongside established declines in cognitive^49^ and physical abilities^50^. Our results suggest that aging societies could have positive impacts nationally but negatively affect international charitable giving. Age was also associated with increased in-group preferences. These findings have important implications for predicting the consequences of population aging and for promoting behaviors that benefit society and protect public health.

## Methods

The preregistration can be found at 10.17605/OSF.IO/9WVP4. Deviations from the preregistered analysis plan are outlined in the Supplementary Methods.

### Participants

Data were collected from participants in 67 countries as part of the International Collaboration on Social & Moral Psychology: COVID-19 project^25^. Ethical approval was obtained from the University of Kent, and the research was in accordance with the World Medical Association Declaration of Helsinki. Participants gave informed consent before starting the survey. Participants were mostly recruited through survey companies and compensated for their time in line with the researchers’ or company’s local policy. A total of 50,944 participants completed the study. The sample size was determined by the number of countries in which researcher teams volunteered to collect data, with a target sample size of 500 participants in each country. At least 500 participants were recruited in 43 countries and 47 collected at least 450 participants (500 – 10% excluded; see below). Some Latin American countries had smaller samples (16–142 per country) so are included when the analysis technique can accommodate different numbers of observations between groups, but not in analysis of each country separately. Research teams aimed to make samples representative of that country’s population in terms of age (over 18) and gender. At the end of data collection, researchers from each team reported whether they had fully achieved representativeness in these terms, and this was the case in 30 countries. Defining representative samples by population age and gender is consistent with the definition of representativeness used by international recruitment companies (for example Luc.id and Prolific.ac) and many global studies that recruit representative samples.

Data were excluded for participants who: did not answer at least 75% of the survey; did not report their age, were aged under 18 or over 100; or failed an attention check. This left a final sample of 46,576 (91.43% of total) that was randomly divided into 2 equal subsamples, S1 and S2, of 23,288 participants each to enable internal replication (mean age (standard deviation) S1 | S2 = 43.00 (15.98) | 43.14 (16.03); 52% | 51% women, 48% men in both subsamples, <1% other genders in both subsamples). We had access to data from 4,587 randomly selected participants in advance of the preregistration. The two subsamples were created pseudorandomly, evenly dividing participants whose data we had access to before the preregistration. We also ran the main models testing the effect of age on both prosocial measures excluding these data (Supplementary Table 2).

A previous study found that students who have experience with economic games may behave differently in a dictator game and students are more likely to be young, so student status could contribute to age differences^51^. A notable strength of our study is that only 11% of our sample were students, much lower than for many psychology studies. To further exclude the possibility that being a student explained the age effects, we also ran control analysis excluding all students and show that results from our three main models are robust (Supplementary Table 10). Another recent study highlighted the possibility that the COVID-19 pandemic could have age-dependent effects on well-being^52^. Although this study found similar age-related advantages in emotional well-being during the pandemic to those found before the pandemic, we ran an additional control analysis to show that any change in well-being over a month of the pandemic was not significantly associated with age (Pearson’s *r* = −0.06, *P* = 0.22; Supplementary Fig. 7; *n* = 448 participants who completed the survey at two time points; see Supplementary Methods).

### Procedure

All participants completed the survey online, with many countries using Qualtrics (Provo, Utah; April 2020) or a similar platform. Where relevant, the survey was translated into the local language using the standard forward–backward translation method. Questionnaires were completed in a random order followed by demographic questions. Unless stated otherwise, all scales were measured on an 11-point (0–10) scale of strongly disagree (0) to strongly agree (10), meaning that high scores are associated with increased levels of the named variable.

Data were collected between 22 April and 30 May 2020 (Supplementary Fig. 1). On the first day of data collection, worldwide there were a total of 2,516,991 recorded COVID-19 cases and 180,098 recorded deaths from the disease, with 85,730 new cases and 7,284 new deaths reported that day. By the last day, total numbers had approximately doubled to 5,777,512 cases and 360,090 deaths, although the rate of new deaths was lower (4,701; Supplementary Fig. 2), despite new case numbers remaining high (118,805). Moreover, at the time of data collection, whether vaccines could be developed was uncertain.

### Prosocial measures

#### Distancing

Distancing was measured through five items assessing the extent to which participants were effortfully changing their behavior to avoid social contact in line with physical distancing measures, on a scale of 0 (strongly disagree) to 10 (strongly agree) of whether “during the last 4 weeks, I have been…” 1) staying at home as much as practically possible, 2) visiting friends, family or colleagues outside my home (reversed), 3) keeping the number of grocery store visits at an absolute minimum, 4) keeping physical distance from all other people outside my home, 5) avoiding handshaking with people outside my home. We excluded item 2 when averaging the scale, in line with reliability analysis of this scale in other work^25^. Data were missing on this measure for 21 | 34 participants in S1 | S2.

#### Charitable donations

Charitable donations were measured using a single item adapted from the dictator game applied to hypothetical charitable donations. Participants were asked to imagine they received an amount of money (the median daily wage in their country) and what percentage they would: keep, donate to a national charity; and donate to an international charity. Each hypothetical charity was described as a charity organization providing medical support for COVID-19 either “in your own country” or “all over the world” respectively. There is evidence that hypothetical dictator game measures are comparable to non-hypothetical measures in terms of amount donated and associations with age^8,9,53^. A hypothetical version for charitable donations, similar to the current measure, was selected for an international survey as it predicted an incentivized measure^27,28,54^. Using a hypothetical measure has important strengths as it enabled using a substantial amount (median daily wage). The fact that more money was kept than donated suggests that participants did not give unrealistic amounts simply because it was hypothetical. Our focus on charities responding to COVID-19 allowed us to have the same cause for both a national and international charity for participants from any country. Analyses were run on the total amount donated, summed across the national and international charity, and donations to each charity separately. Donation amounts had a trimodal distribution so were logit transformed to better meet normality assumptions, as previously applied to dictator game data^55^. Donation amounts in the text and plots are raw percentages to aid interpretation. No participants in S1 and one in S2 were missing this data.

### Control variables

#### Perceived risk

Perceived risk was measured using a probability rating (0–100%) of how likely it is “you will get the Coronavirus by the 30th of April 2021” (in approximately a year, date standardized across testing so time until that date varied slightly). Data were missing on this measure for 97 participants in S1 and 114 in S2.

#### Physical health

Physical health was measured using a single item asking, “In general, how would you rate your physical health as it is today?” rated from 0 (extremely bad) to 10 (extremely good). A total of 118 participants and 129 participants in S1 and S2, respectively, were missing data.

#### Subjective wealth

Subjective wealth was measured according to where on an 11-point “ladder” people see themselves compared to other people in their country: with 10 being the people who are the best off–those who have the most money, the most education and the most respected jobs, and 0 being the people who are the worst off—who have the least money, least education and the least respected jobs or no jobs. A total of 32 participants in S1 and 29 in S2 had missing data.

#### Objective wealth

Objective wealth was based on self-reported income in 5 countries with these data (UK, Nigeria, the Philippines, Singapore and Ukraine; *n* = 2,624), used in control analyses. Data were cleaned to remove non-numeric characters, and if participants had entered a range (for example, 1,000–2,000), the midpoint was used. Values were then *z*-scored for each country separately.

#### Gender

Gender was recorded as a binary factor of male or female for participants who reported this. Responses of ‘other’ were not included in analysis as there were not enough responses (<1%) to model differences between three levels.

#### Adjusted age

Adjusted age was determined using raw age rescaled to a proportion of life expectancy for the relevant gender in the participants’ country^56^. If no gender or a non-binary gender was reported, the overall life expectancy was used.

### Country-level variables

#### COVID-19 severity

We quantified severity with the number of deaths as this is salient, widely reported and likely to be more consistent across time and countries than case numbers, which depend more on testing capacity. Two statistics were calculated using data from Global Change Data Lab^57^. The first is the total number of deaths recorded up to the day before the participant completed the study, log_10_ transformed as the raw distribution was heavily skewed. The second is the rate of new deaths calculated as the coefficient from a regression over the number of new deaths per day (7-day rolling average) in the 7 days before the participant completed the study. For this measure, a positive value indicates that the rate of deaths was increasing and a negative value means the rate was decreasing. We calculated each of these statistics both in the participant’s own country and worldwide meaning there are four measures of COVID-19 deaths: total worldwide; rate worldwide; total in country; and rate in country. Data were matched to the relevant day using custom scripts in MATLAB 2019b (The MathWorks Inc). This required the exact date of data collection for each participant, and this was missing in 26 countries. Although the start and end dates of data collection were available (Supplementary Fig. 1), the situation was changing so quickly it did not seem appropriate to use a proxy for the date of survey completion. Thirteen of these countries were those with sample sizes less than 100, but a total of 3,486 | 3,487 participants in S1 | S2 lack COVID-19 deaths data due to unknown dates of survey completion.

#### Country wealth

Country wealth was measured as gross national income (GNI) per capita in 2018 from the World Bank^58^. Data were not available for Taiwan (not included in China GNI, but not reported separately), Cuba (last available data 2016) or Venezuela (last available data 2014). This meant that 475 participants in S1 and 482 participants in S2 were missing country wealth data.

### Socioemotional traits

#### Collective narcissism

Collective narcissism was measured using an ultrashort three-item version with statements such as “[My national group] deserves special treatment”^59^. For each country, the statements applied to the relevant “national group”.

#### National identification

National identification was measured using two items applied to the local “nationality”: “I identify as [nationality]”^60^ and “Being a [nationality] is an important reflection of who I am”.

#### Political ideology

Political ideology was measured as a single item, “Overall, what would be the best description of your political views?”, on an 11-point scale from very left-leaning (0) to very right-leaning (10)^61^. These terms were chosen to be more generalizable to multiple political systems than ‘liberal’ and ‘conservative’, but the meanings of ‘left’ and ‘right’ do still vary between cultures and countries^62^.

#### Narcissism

Narcissism was measured using six items; for example, “I deserve to be seen as a great personality”^63^.

#### Open-mindedness

Open-mindedness was measured on a six-item scale assessing intellectual humility^64^, with an example item: “I feel no shame learning from someone who knows more than me”.

#### Moral identity

Moral identity was measured using ten items assessing identification with being a person with the characteristics “caring, compassionate, fair, friendly, generous, helpful, hardworking, honest, kind”^65^.

#### Moral circle

The extent of participants’ moral circle was measured using a single item asking for “the circle of people or other entities for which you are concerned about right and wrong done toward them”^66^ to be indicated on a 16-point scale ranging from a small moral circle (1—all of your immediate family) to a large moral circle (16—all things in existence).

#### Well-being

Well-being was measured through two items: “In general, to what extent do you feel happy these days?” (0 = very unhappy, 10 = very happy) and Cantril’s Ladder rating one’s current life on a scale from worst (0) to best (10) possible life (adapted from the World Happiness Report and the Gallup World Poll; see Bjørnskov^67^).

#### Optimism

Optimism was measured through two items: “As a person, I am optimistic for my future” and “Overall, I expect more good things to happen to me than bad”^68^.

#### Social belonging

Social belonging was measured through four items with an example item: “I feel accepted by others”^69^.

#### Self-control

Self-control was measured on a four-item scale; for example, “I am good at resisting temptation”^70^.

#### Self-esteem

Self-esteem was measured through a single item: “I have high self-esteem”^71^.

#### Morality-as-cooperation

Morality-as-cooperation was measured through seven items on the extent to which a moral consideration is relevant to deciding “whether something is right or wrong”^72^. Each item relates to a separate moral domain: family (“helped a member of their family”), group (“worked to unite their community”), reciprocity (“kept their promise”), heroism (“showed courage in the face of adversity”), deference (“deferred to those in authority”), fairness (“kept the best part for themselves”) and property (“kept something that didn’t belong to them”).

### Statistics

We ran all analyses^34^ in R (ref. ^73^; v3.6.2) with R Studio^74^ (v1.4.1106). CIs around means for binary factors (for example, international versus national charity) were calculated using a bootstrapping method (mean_cl_boot function from ggplot2 package^75^). LMMs were fitted to each subsample separately using the lme4 package^76^. Degrees of freedom were calculated using the Satterthwaite method^77^. As specified in our preregistration, the significance threshold for fixed effects from LMMs on each subsample was set to *P* *<* 0.01. Standardized regression coefficients (*β*) were calculated using the parameters package (model_parameters function^78^). For binary factors, we report Cohen’s *d* effect sizes calculated from model *t*-values (t_to_d function from effectsize package^79^).

#### Models of distancing and donations

To test our main hypotheses, we ran LMMs predicting distancing and donations. All variables, including logit-transformed donation amounts, were *z*-scored before inclusion in models. All models included a fixed effect of gender (binary factor; men = 0, women = 1) and a fixed effect of the relevant control variable (perceived risk for distancing, subjective wealth for donations). The random effects were uncorrelated country-level intercepts and random slopes of age and subjective wealth. Quadratic fixed effects of age were raw, not orthogonalized, but calculated after *z*-scoring age, which prevented multicollinearity.

#### Country-level analyses

To assess whether the effects of age on prosocial behavior were similar across countries, we fitted three linear models to each country’s data: predicting distancing, controlling for gender and perceived risk; predicting national donations, controlling for gender and subjective wealth; and predicting international donations, controlling for gender and subjective wealth. As not all countries reached the recruitment target of 500 participants, we maintained power by combining subsamples. To assess whether effects changed over time, we also ran these separate models on the data from each separate day of data collection, across all countries, on days that more than 100 participants completed the study. These models were also fitted on all the data, not divided into subsamples. We then extracted standardized *β* coefficients to plot on maps (Fig. 2) and over time (Supplementary Figs. 1 and 2). We also assessed whether country-level variables—country wealth and four measures of COVID-19 deaths—moderated the effects of age on distancing and donating or the interaction between age and country location (international versus national). Total deaths were log_10_ transformed then these numbers, the death rates and GNI were *z*-scored, and entered as additional fixed effects and interactions in LMMs. For models of distancing, we included a main effect of each country-level variable and the five two-way interactions between these and age. For models of donations, we started with a maximal model that included all three-way interactions between country-level variables, age and charity location (international versus national) plus all two-way interactions with age and with charity locations. We then reduced this model by removing terms, starting with the three-way interactions, if they did not significantly improve the model fit (*χ*^2^ test using anova function with a *P* < 0.05 threshold) in both subsamples. Correlations between our five country-level variables (four COVID-19 death measures and wealth), age and the individual-level control variables (perceived risk and subjective wealth) were all low enough that multicollinearity was not an issue (*r* values < 0.46 in S1 and S2).

#### Factor analysis

We first ran a parallel analysis with the psych package^80^ (fa.parallel function) to determine the number of factors from the data. All 19 of the socioemotional traits were entered and participants were included only if they had complete data on all measures (*n* = 22,253 | 22,275). For both subsamples, the parallel analysis suggested a six-factor solution. We then ran the factor analysis with the psych package (fa function) using ordinary least squares to find the minimum residual solution (minres method) as recommended for this package. Solutions using both an oblique rotation (oblimin) and orthogonal rotation (varimax) were examined and produced similar factor loadings. Several pairs of factors had correlations of approximately 0.30, but based on the nature of the measures, there was no strong reason to expect orthogonalization. Using an oblique rotation also eliminated cross-loading based on a threshold of 0.30, so we report results from this analysis. Due to the substantial sample size, only factor loadings of 0.30 and above are reported. In S2, the general morality factor consisted of moral identity and moral circle. In S1, moral circle did not load onto any factor above 0.30, so the only measure for general morality was moral identity. However, the loading for moral circle onto this factor in S1 was 0.27, close to the 0.30 threshold. This suggests the underlying factor was similar, and so the six-factor structure was maintained in both subsamples for consistency. Participant-level scores on each of the six factors were calculated using the regression method within the factor analysis.

#### Factor correlations and structural equation models

We ran Pearson’s correlations between each factor and age, distancing and donations to each charity and then used the psych package^80^ to test whether pairs of correlations were significantly different (exploratory analysis; *P* < 0.0001 Bonferroni-corrected) from each other (paired.r function). Finally we used the lavaan package^81^ for structural equation models (see Supplementary Methods for details).

### Reporting Summary

Further information on research design is available in the Nature Research Reporting Summary linked to this article.

## Supplementary information

Supplementary Information Supplementary Methods, Results, Figs. 1–5 and Tables 1–7.

Reporting Summary

## Data Availability

The survey data are available from the International Collaboration on Social & Moral Psychology: COVID-19 website: https://icsmp-covid19.netlify.app/. Data on COVID-19 rates are available from the Global Change Data Lab Our World in Data website: https://ourworldindata.org/coronavirus. Life expectancy and gross national income data are available from Worldometer: https://www.worldometers.info/demographics/life-expectancy/ and the World Bank: https://databank.worldbank.org/source/world-development-indicators, respectively. The full, specific dataset used in this study and the preregistration are available from 10.17605/OSF.IO/9WVP4. Source data are provided with this paper.

## Source data

Source Data Fig. 1 Statistical Source Data.
Source Data Fig. 2 Statistical Source Data.
Source Data Fig. 3 Statistical Source Data.
Source Data Fig. 4 Statistical Source Data.

## Code availability

Analysis scripts are available from 10.17605/OSF.IO/9WVP4.
