# Supplementary material for: Older adults across the globe exhibit increased prosocial behavior but also greater in-group preferences
Source: Nat Aging. 2021 Oct 11;1(10):880–8. doi: 10.1038/s43587-021-00118-3 (PMC10154238; doi:10.1038/s43587-021-00118-3)
Supplement: Supplementary file 1 — Supplementary Methods, Results, Figs. 1–5 and Tables 1–7. [file 43587_2021_118_MOESM1_ESM.pdf]

nature portfolio

https://doi.org/10.1038/s43587-021-00118-3

Supplementary information

Older adults across the globe exhibit increased prosocial behavior but also greater in-group preferences

In the format provided by the authors and unedited

Supplementary Information:

Deviations from preregistration

We initially planned to use generalised mixed-effects models with an appropriate link function for analysis of donation amounts. However, no appropriate link function was identified for the trimodal distribution and a logit transformation was judged to be more suitable, especially given that linear mixed-effects models (LMMs) are relatively robust to violations of normality[1]. Our method is also in line with previous use of the logit[2] or similar arcsine-square root[3] transformation for data from the dictator game, which our charitable donations measure approximated. Finally, analysing the logit-transformed values allowed us to use linear models for effects in each country. For the LMM of donations with the national vs. international factor, the random intercept of participant, nested within country, lead to convergence issues, as the participant intercept term did not capture much variance. We removed this term leaving uncorrelated random country-level intercept and slopes of age and subjective wealth. Our description of this model also did not explicitly specify controlling for wealth, as we specified for the model predicting total donations (regardless of national vs. international). Adding this control significantly improved the model in both subsamples and results did not change (see Supplementary Results below) so we report the results controlling for subjective wealth in the main text.

We originally specified that the factor analysis would be across all participants (and not subsamples). However, the final sample size with all measures for the factor analysis was substantial and allowed us to analyse the two subsamples separately. Results showed almost identical factor structures and loadings, thus demonstrating robustness of our findings. For correlations and structural equation models using factor scores, and for patterns in each country, we considered amounts given to national and international charities separately, rather than looking at the total amount donated and bias toward national charities. This was to make results easier to interpret, given the relationship between age and donations was in opposite directions for national and international donations. For the same reason, in the LMM with country-level variables, we added three-way interactions between the country-level variable, age and charity location (national vs. international). Our preregistration also stated we would test whether the individual difference factors “mediated” the effect of age on prosocial behaviour, but we recognise there are issues around interpreting results from cross-sectional data as showing causal mediation[4–7]. While the temporal structure of the variables in our

structural equation models result in unidirectional paths in all cases (see Methods; Fig. S6), we do not use the term "mediate" when discussing the indirect effects. Finally, due to the large sample size and power, we used a p<0.01 Bonferroni-corrected threshold, rather than a familywise detection rate correction with p<0.05 for the analysis of traits. This was applied in each subsample for each group of tests (correlations and each category of path in the structural equation models). For tests of differences between correlations, not included in the preregistration, we made this more stringent at p<0.0001 Bonferroni-corrected.

Test-retest reliability analysis

A sample of 448 participants in the UK completed the measures at two time points, one month apart. Data from the first time point is included in the main analysis. We examined test-retest reliability of the two prosocial measures, distancing and overall donations, as well as the 19 individual difference measures through the intraclass correlation coefficient (ICC) between responses at time 1 and time 2. We calculated ICC using the icc function from the irr package[8] and running a two-way model estimating agreement between time points. Unlike a Pearman's or Spearman's correlation, this measures whether absolute scores remain the same, as well as the relationship or ranking between participants, and includes random effects of participant and time point, so is recommended for test-retest reliability[9].

Factor correlations and structural equation models

For each factor, we calculated the Pearson's correlation with age, distancing, national donations, and international donations. This measure was used despite deviations from normality for some variables due to the large sample and stringent correction to significance levels: we applied a Bonferroni correction with a p<0.01 threshold. We then used structural equation models implemented in the lavaan package[81] to test potential statistical indirect effects of age on distancing, national donations, and international donations via the trait factors. These models allow us to bring the three measures of age, traits, and prosocial behaviour together in a single analysis, estimating the direct and indirect associations concurrently and enabling the traits to be both predicted by age and predict prosocial behaviour. As the independent variables of interest in our models were age and socio-emotional traits, these cannot be experimentally manipulated. However, the directions of effects in our models are based on a logical temporal structure from the fact that no other variable can affect chronological age. Traits that are generally stable across months (see

Table S7 for test-retest reliability over one month) and existed before the pandemic precede the prosocial behaviours, which applied specifically to the recent pandemic context. We ran a structural equation model for each of the three prosocial outcomes: distancing, national donations, and international donations. Each had a direct path from age to the prosocial measure, indirect paths via each trait factor, and the relevant control variable predicting prosocial behaviour (perceived risk for distancing and subjective wealth for both types of donations). Donation amounts were logit transformed and all variables were z-scored as in the main models. Covariances between the trait factors were also included in the model. For each path, we extracted the standardised coefficients and associated *p* values, and Bonferroni corrected the *p* values across all paths for all three structural equation models in each subsample.

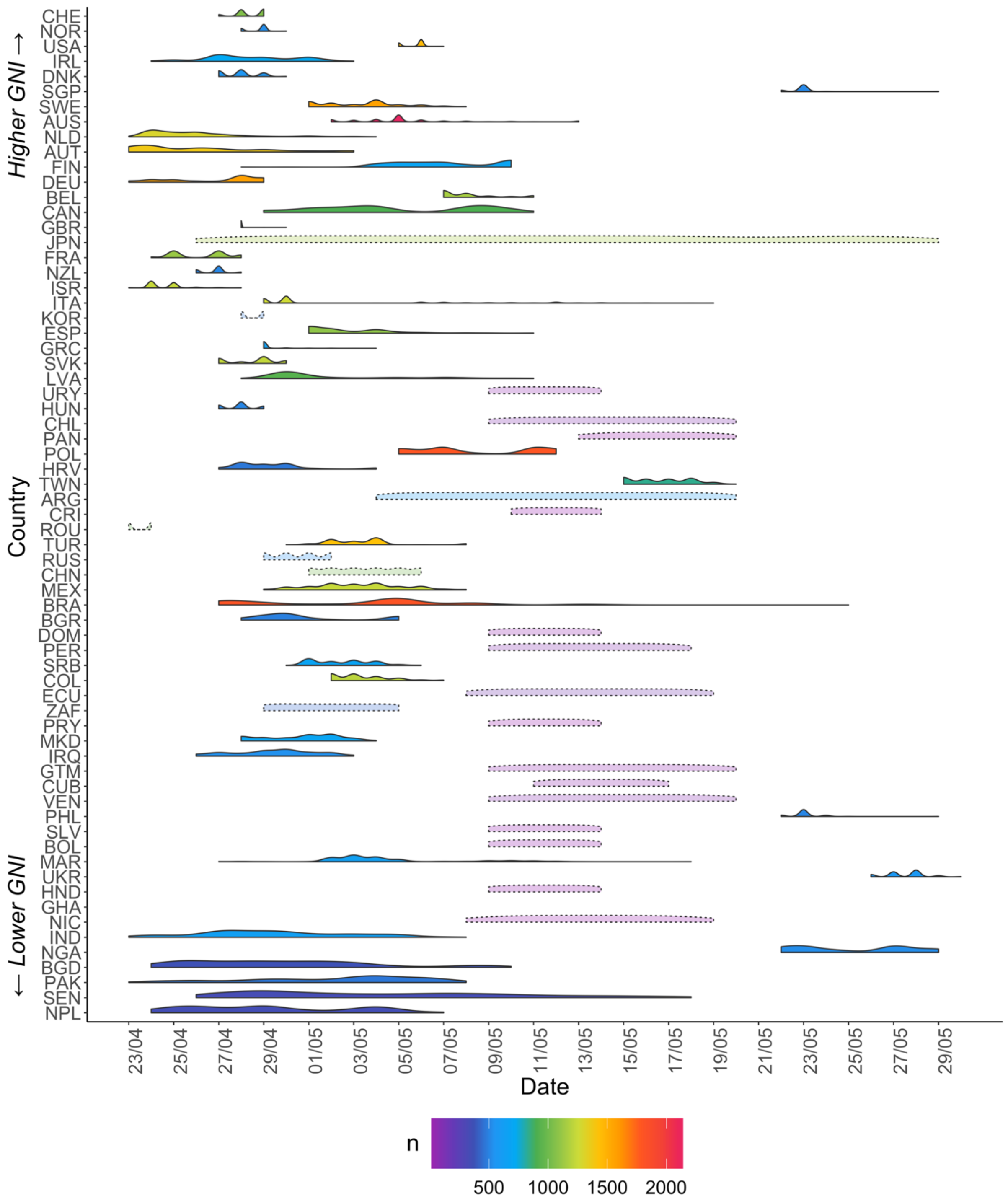

Fig. S1. Data collection periods in each country. Distributions represent the date participants completed the survey. Distributions are relative to the country's sample and the colour shows the sample size. Countries with faded distributions and dashed outlines were missing the date of survey completion for each participant so distributions are just from the start date to the end date of data collection. Countries are labelled with their ISO3 code and sorted by gross national income (GNI) from richest to poorest. Exact GNI was not available for three countries so they are included at the lowest point of their GNI income group (Taiwan – TWN: high income; Venezuela – VEN: upper-middle income; Cuba – CUB: upper-middle income).

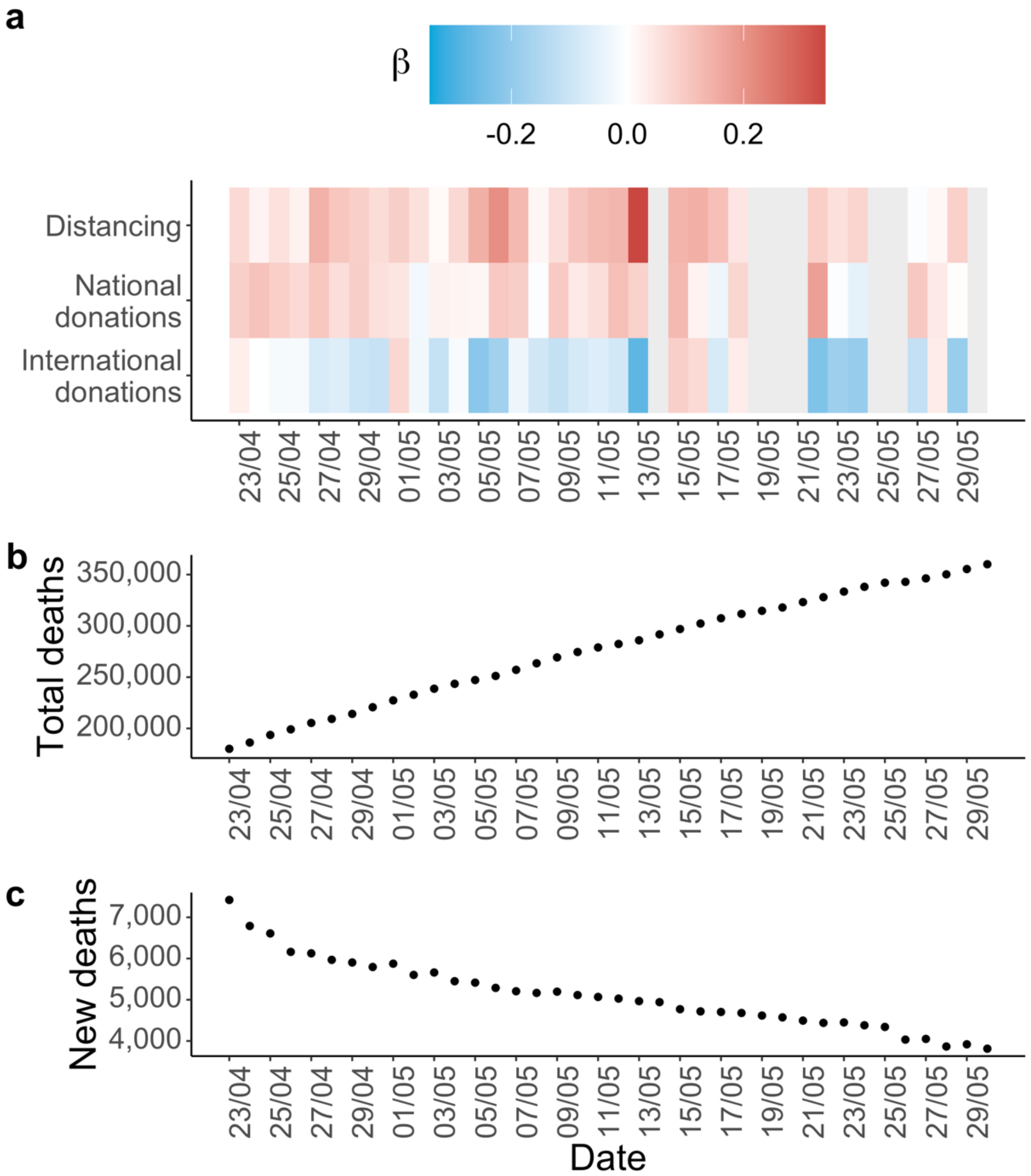

Fig. S2. Age effects on prosocial behaviour and COVID-19 deaths over the period of data collection. (**a**) Standardised regression βs for the effect of age on each prosocial outcome measure were calculated on each day of data collection that more than 100 people completed the survey on. βs do not show a systematic relationship with time, including relative to when countries of differing wealth collected data (see Fig. S1). (**b**) The total number of deaths from COVID-19 rose steadily over the time data were being collected. (**c**) The rate of new deaths each day (7-day rolling average) from COVID-19 worldwide fell over the period of data collection.

Supplementary Results

Results are the same when excluding the 10% of data available for preliminary analysis

Before our preregistration, we received a randomly selected 10% of the overall dataset and ran preliminary analyses relevant to the preregistered hypotheses. These data were included in the full dataset to increase power, particularly for country-level effects. Participants whose data were in this 10% were divided evenly between the two subsamples. We also showed that the key findings are the same when excluding these participants (see Table S2).

Age effects are not driven by differences between countries in the range of ages in the population

Results in the main text are reported from analyses using participants' raw age as predictors. We also tested whether key findings remained the same when using participants' age adjusted to be a proportion of the life expectancy in the relevant country. When participants reported their gender as male or female, the life expectancy for their gender was used. A value of 1 on this scale represents an age equal to the average life expectancy. If participants reported having a non-binary gender or did not report their gender, the life expectancy for the whole population in the country was used. All results reported in the main text for raw age were the same when using age adjusted for life expectancy (Table S3). Adjusted age predicted higher distancing scores, larger overall donations, and more national bias in giving.

Table S1.

Results from linear mixed-effects models predicting distancing and hypothetical charitable donations (total and by charity location)

	Subsample 1						Subsample 2					
**Distancing**	β	CI low	CI up	*t*	df	*p*	β	CI low	CI up	*t*	df	*p*
Intercept	-0.11	-0.18	-0.04	-3.13	72.77	0.003	-0.09	-0.16	-0.02	-2.61	71.87	0.01
Gender (F > M)	0.27	0.25	0.30	21.49	23058.13	<0.001	0.28	0.25	0.30	21.62	23038.14	<0.001
Perceived risk	0.02	0.00	0.05	1.83	53.13	0.07	0.02	0.00	0.05	1.69	50.88	0.10
Age	0.10	0.07	0.13	6.29	56.27	<0.001	0.10	0.07	0.13	6.74	50.98	<0.001
**Total donations**												
Intercept	-0.10	-0.18	-0.03	-2.76	74.88	0.01	-0.08	-0.15	0.00	-2.06	75.88	0.04
Gender (F > M)	0.14	0.12	0.17	11.34	23021.19	<0.001	0.16	0.13	0.18	12.36	23042.42	<0.001
Subjective wealth	-0.08	-0.10	-0.06	-8.78	39.46	<0.001	-0.08	-0.11	-0.06	-6.76	52.41	<0.001
Age (linear)	0.04	0.02	0.06	4.29	55.68	<0.001	0.05	0.03	0.07	5.00	46.19	<0.001
Age (quadratic)	0.06	0.04	0.07	8.56	6780.73	<0.001	0.04	0.02	0.05	5.71	6244.72	<0.001
**Donations by charity**												
Intercept	0.29	0.24	0.34	11.59	74.38	<0.001	0.30	0.25	0.35	11.57	74.63	<0.001
Gender (F > M)	0.10	0.08	0.12	11.60	45359.41	<0.001	0.12	0.10	0.13	13.20	45684.43	<0.001
Subjective wealth	-0.05	-0.06	-0.04	-8.38	48.54	<0.001	-0.05	-0.07	-0.04	-6.30	52.12	<0.001
Age	0.08	0.06	0.09	8.09	72.78	<0.001	0.07	0.05	0.09	7.65	67.90	<0.001
Charity (I > N)	-0.66	-0.67	-0.64	-76.60	45798.29	<0.001	-0.66	-0.68	-0.64	-77.00	45827.23	<0.001
Age * Charity	-0.12	-0.14	-0.11	-14.28	45798.74	<0.001	-0.11	-0.12	-0.09	-12.63	45827.79	<0.001

Note. CI low / up: 95% confidence interval lower / upper, F: female, M: male, I: international charity, N: national charity.

Table S2.

Results from linear mixed-effects models excluding the 10% of participants in preliminary analysis

	**Subsample 1**						**Subsample 2**					
**Distancing**	β	CI low	CI up	*t*	df	*p*	β	CI low	CI up	*t*	df	*p*
Intercept	-0.11	-0.18	-0.03	-2.91	72.39	0.005	-0.09	-0.16	-0.02	-2.39	71.64	0.02
Gender (F > M)	0.27	0.24	0.30	20.07	20776.84	<0.001	0.27	0.25	0.30	20.21	20770.87	<0.001
Perceived risk	0.02	0.00	0.05	1.69	52.75	0.10	0.03	0.00	0.05	2.11	48.80	0.04
Age	0.10	0.07	0.14	6.26	54.24	<0.001	0.10	0.07	0.13	6.85	49.86	<0.001
**Total donations**												
Intercept	-0.10	-0.17	-0.03	-2.62	76.03	0.01	-0.08	-0.16	-0.01	-2.15	75.36	0.03
Gender (F > M)	0.14	0.11	0.17	10.44	20749.65	<0.001	0.15	0.13	0.18	11.60	20756.26	<0.001
Subjective wealth	-0.08	-0.10	-0.06	-8.62	37.83	<0.001	-0.08	-0.11	-0.06	-6.73	50.62	<0.001
Age (linear)	0.04	0.02	0.06	3.95	58.52	<0.001	0.04	0.03	0.06	4.55	44.83	<0.001
Age (quadratic)	0.06	0.04	0.07	8.10	6399.49	<0.001	0.04	0.03	0.05	5.87	5488.07	<0.001
**Donations by charity**												
Intercept	0.29	0.24	0.34	11.68	75.88	<0.001	0.29	0.24	0.35	11.15	74.15	<0.001
Gender (F > M)	0.10	0.08	0.12	10.67	40741.74	<0.001	0.11	0.10	0.13	12.41	41095.13	<0.001
Subjective wealth	-0.05	-0.07	-0.04	-8.42	47.10	<0.001	-0.05	-0.07	-0.04	-6.35	49.67	<0.001
Age	0.07	0.06	0.09	7.92	76.77	<0.001	0.07	0.05	0.09	7.10	66.89	<0.001
Charity (I > N)	-0.66	-0.67	-0.64	-72.72	41243.08	<0.001	-0.65	-0.67	-0.64	-72.60	41258.32	<0.001
Age * Charity	-0.12	-0.14	-0.11	-13.68	41243.59	<0.001	-0.11	-0.12	-0.09	-11.75	41258.94	<0.001

Note. CI low / up: 95% confidence interval lower / upper, F: female, M: male, I: international charity, N: national charity.

Table S3.

Results from linear mixed-effects models using age adjusted to a proportion of the participants' country life expectancy

	**Subsample 1**						**Subsample 2**					
**Distancing**	beta	CI low	CI up	*t*	df	*p*	beta	CI low	CI up	*t*	df	*p*
Intercept	-0.11	-0.18	-0.05	-3.27	72.68	0.002	-0.1	-0.16	-0.03	-2.79	71.83	0.007
Gender (F > M)	0.27	0.25	0.30	21.51	23057.37	<0.001	0.28	0.25	0.30	21.63	23037.49	<0.001
Perceived risk	0.02	0.00	0.05	1.83	53.18	0.07	0.02	0.00	0.05	1.71	51.05	0.09
Adjusted age	0.10	0.07	0.13	6.23	57.38	<0.001	0.10	0.07	0.13	6.73	53.19	<0.001
**Total donations**												
Intercept	-0.10	-0.17	-0.03	-2.65	74.78	0.01	-0.08	-0.15	0.00	-2.04	76.02	0.05
Gender (F > M)	0.14	0.12	0.17	11.32	23049.57	<0.001	0.16	0.13	0.18	12.35	23060.32	<0.001
Subjective wealth	-0.08	-0.10	-0.06	-8.74	39.39	<0.001	-0.08	-0.11	-0.06	-6.72	52.45	<0.001
Adj. age (linear)	0.04	0.02	0.06	3.96	50.85	<0.001	0.05	0.03	0.07	4.73	43.91	<0.001
Adj. age (quadratic)	0.05	0.04	0.06	7.59	8432.62	<0.001	0.03	0.02	0.05	5.12	7849.33	<0.001
**Donations by charity**												
Intercept	0.29	0.24	0.34	11.62	74.97	<0.001	0.3	0.25	0.35	11.67	75.14	<0.001
Gender (F > M)	0.10	0.08	0.12	11.61	45350.16	<0.001	0.12	0.10	0.13	13.21	45682.61	<0.001
Subjective wealth	-0.05	-0.06	-0.04	-8.38	48.54	<0.001	-0.05	-0.07	-0.04	-6.30	52.13	<0.001
Adjusted age	0.08	0.07	0.10	9.26	80.10	<0.001	0.08	0.06	0.10	8.70	72.62	<0.001
Charity (I > N)	-0.66	-0.67	-0.64	-76.65	45796.52	<0.001	-0.66	-0.68	-0.64	-77.05	45826.26	<0.001
Adj. age * Charity	-0.14	-0.16	-0.12	-16.20	45797.02	<0.001	-0.12	-0.14	-0.11	-14.52	45827.00	<0.001

Note. CI low / up: 95% confidence interval lower / upper, F: female, M: male, Adj.: Adjusted, I: international charity, N: national charity.

Table S4.

Results from linear mixed-effects models of distancing controlling for participants' self-reported physical health condition

	Subsample 1						Subsample 2					
**Distancing**	beta	CI low	CI up	*t*	df	*p*	beta	CI low	CI up	*t*	df	*p*
Intercept	-0.12	-0.18	-0.05	-3.38	73.02	0.001	-0.1	-0.16	-0.03	-2.82	72.11	0.006
Gender (F > M)	0.28	0.25	0.30	21.74	22964.50	<0.001	0.28	0.25	0.30	21.75	22919.86	<0.001
Perceived risk	0.03	0.00	0.05	2.22	52.83	0.03	0.02	0.00	0.05	2.10	49.46	0.04
Health condition	0.06	0.03	0.08	4.67	49.95	<0.001	0.05	0.02	0.07	3.98	54.69	<0.001
Age	0.10	0.07	0.14	6.58	55.88	<0.001	0.10	0.07	0.13	6.96	50.47	<0.001

Note. CI low / up: 95% confidence interval lower / upper, F: female, M: male.

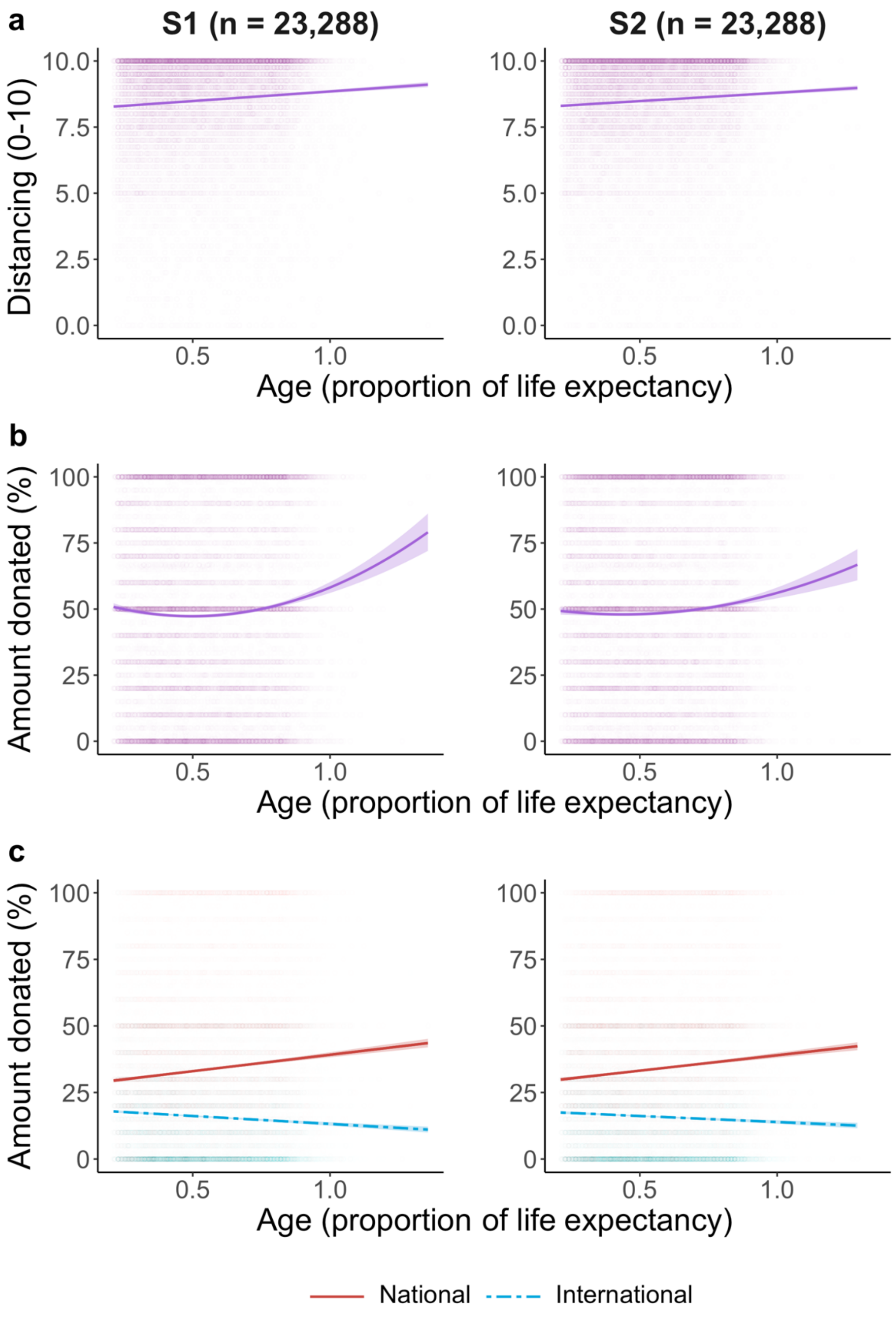

Fig. S3. Age adjusted for life expectancy is associated with prosocial behaviour. In both subsample 1 (left) and subsample 2 (right), age as the proportion of life expectancy in the participants' country predicted greater prosocial behaviour. On this scale, 1 represents an age equal to the average life expectancy. **(a)** Older age predicted higher rates of distancing and **(b)** hypothetical charitable donations when summed across both charities. The relationship between age and total donations is quadratic. **(c)** Age was positively associated with donations to a national charity but negatively associated with donations to an international charity. Lines show fitted linear models, shaded areas show 95% confidence intervals.

The relationship between age and donations to national & international charities is robust with or without controlling for subjective wealth

As described in the deviations from the preregistration, we included a fixed and a random effect of subjective wealth as a control variable in the model predicting donations to national and international charities separately. For both subsamples, this significantly improved the model fit ($\chi^2(2)$ S1 | S2: 177 | 225, $p<0.001$ $|<0.001$) so the results from this model are reported within the text. Without controlling for subjective wealth, the key predictors remained significant such that older people gave more overall (β=0.08 | 0.07, $p<0.001$ $|<0.001$) and this relationship was significantly less positive (in fact negative) for international, compared to national, charities (interaction β=−0.12 | −0.11, $p<0.001$ $|<0.001$).

Age is associated with increased donations and national bias in giving when controlling for objective wealth

In a subset of participants (n=2,624, 5 countries) where we had objective wealth data (monthly income, see Methods) we ran an additional control analysis to assess whether the relationship between age and national/international donations remained. Due to the smaller sample, we analysed the data as one group and applied a $p<0.05$ threshold. As reported in the main text, after controlling for objective wealth, age was still positively associated with donations (β=0.11, $p=0.02$; Table S5 & Fig. S4) and greater bias towards national charities (interaction β=−0.22, $p<0.001$). The effects of charity location (international vs. national d=−0.78, $p<0.001$) and gender (female vs. male d=0.14, $p<0.001$) also remained significant and had sizes comparable to the full sample. Unlike subjective wealth, objective wealth did not significantly predict donations ($p=0.45$).

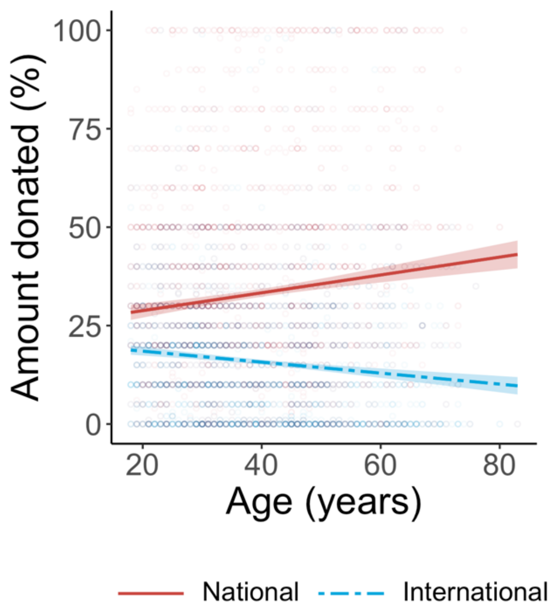

Fig. S4. The relationship between age and donations in the participants with objective wealth data. Data on objective wealth (self-reported monthly income) was available from 2624 participants in 5 countries (UK, Singapore, Nigeria, the Philippines, and Ukraine). Age was associated with increased donations to the national charity but decreased donations to the international charity, as in the full dataset. Lines show fitted linear models, shaded areas show 95% confidence intervals.

Table S5.

Results from linear mixed-effects model predicting donations by charity controlling for objective wealth (n=2624, 5 countries)

	β	CI low	CI up	*t*	df	*p*
Intercept	0.28	0.13	0.44	3.51	4.41	0.02
Gender (F > M)	0.13	0.08	0.18	4.98	5199.67	<0.001
Objective wealth	0.02	-0.03	0.06	0.83	4.19	0.45
Age	0.11	0.04	0.18	3.10	5.51	0.02
Charity (I > N)	-0.71	-0.76	-0.66	-28.20	5220.30	<0.001
Age * Charity	-0.22	-0.27	-0.17	-8.65	5220.30	<0.001

Note. CI low / up: 95% confidence interval lower / upper, F: female, M: male, I: international charity, N: national charity.

Table S6.
Results from linear mixed-effects models including country-level variables

	Subsample 1						Subsample 2					
**Distancing**	β	CI low	CI up	*t*	df	*p*	β	CI low	CI up	*t*	df	*p*
Intercept	-0.14	-0.21	-0.06	-3.49	42.47	0.001	-0.13	-0.20	-0.05	-3.41	42.74	0.001
Gender (F > M)	0.29	0.26	0.31	20.33	19168.04	<0.001	0.29	0.26	0.31	20.34	19167.83	<0.001
Perceived risk	0.03	0.02	0.04	4.01	19151.64	<0.001	0.03	0.01	0.04	3.69	19112.64	<0.001
Age	0.12	0.09	0.15	7.01	33.24	<0.001	0.11	0.07	0.14	6.00	28.99	<0.001
Deaths - DTW	-0.04	-0.07	0.00	-2.01	756.29	0.04	-0.05	-0.08	-0.01	-2.61	674.84	0.009
Deaths - DRW	-0.02	-0.04	0.01	-1.50	4560.54	0.13	-0.03	-0.05	0.00	-2.34	3900.96	0.02
Deaths - DTC	0.09	0.01	0.17	2.19	46.96	0.03	0.09	0.02	0.17	2.47	47.25	0.02
Deaths - DRC	0.03	-0.02	0.08	1.23	526.61	0.22	0.06	0.01	0.11	2.26	449.79	0.02
Country wealth	-0.10	-0.17	-0.02	-2.58	40.92	0.01	-0.11	-0.18	-0.04	-2.99	41.23	0.005
Age * DTW	0.00	-0.02	0.03	0.25	184.95	0.80	0.01	-0.02	0.03	0.51	168.33	0.61
Age * DRW	0.01	-0.02	0.03	0.47	559.95	0.64	0.02	0.00	0.04	1.62	543.37	0.11
Age * DTC	-0.01	-0.05	0.03	-0.67	39.63	0.51	-0.01	-0.05	0.03	-0.46	35.42	0.65
Age * DRC	-0.02	-0.06	0.01	-1.39	63.39	0.17	-0.01	-0.05	0.02	-0.67	60.23	0.50
Age * CW	0.05	0.01	0.08	2.58	36.57	0.01	0.04	0.00	0.07	1.91	32.35	0.07
**Donations by charity**												
Intercept	0.27	0.23	0.32	11.57	45.82	<0.001	0.27	0.22	0.32	10.18	44.55	<0.001
Gender (F > M)	0.11	0.09	0.13	11.56	37995.17	<0.001	0.12	0.11	0.14	13.04	38231.10	<0.001
Subjective wealth	-0.06	-0.07	-0.04	-8.03	39.34	<0.001	-0.05	-0.07	-0.04	-6.35	39.38	<0.001
Age	0.10	0.08	0.12	10.62	68.98	<0.001	0.09	0.07	0.11	9.29	59.45	<0.001
Charity (I > N)	-0.67	-0.68	-0.65	-70.59	38426.60	<0.001	-0.66	-0.68	-0.64	-70.69	38435.12	<0.001
Deaths - DTW	-0.03	-0.06	-0.01	-2.70	738.26	0.007	-0.04	-0.07	-0.02	-3.64	957.46	<0.001
Deaths - DTC	0.00	-0.05	0.04	-0.20	49.61	0.84	0.01	-0.05	0.06	0.26	48.71	0.80
Deaths - DRC	0.04	0.00	0.07	2.22	570.46	0.03	0.04	0.01	0.08	2.39	809.75	0.02

Country wealth	-0.18	-0.22	-0.13	-7.64	44.19	<0.001	-0.17	-0.22	-0.12	-6.54	43.61	<0.001
Age * Charity	-0.17	-0.19	-0.15	-17.68	38426.60	<0.001	-0.15	-0.16	-0.13	-15.06	38435.12	<0.001
Age * DTW	-0.02	-0.03	0.00	-2.56	127.88	0.01	-0.01	-0.03	0.00	-1.93	111.36	0.06
Charity * DTW	0.05	0.04	0.07	5.69	38426.60	<0.001	0.06	0.04	0.08	5.89	38435.12	<0.001
Age * DTC	0.00	-0.03	0.02	-0.41	72.84	0.68	-0.01	-0.03	0.01	-1.23	64.61	0.22
Charity * DTC	0.06	0.04	0.08	5.22	38426.60	<0.001	0.04	0.02	0.06	3.99	38435.12	<0.001
Age * DRC	-0.02	-0.04	0.00	-1.97	87.34	0.05	-0.03	-0.05	-0.01	-2.53	75.29	0.01
Charity * DRC	-0.02	-0.05	0.00	-2.32	38426.60	0.02	-0.03	-0.05	0.00	-2.39	38435.12	0.02
Age * CW	0.02	0.00	0.04	2.50	42.14	0.02	0.02	0.00	0.04	2.31	36.73	0.03
Charity * CW	0.14	0.12	0.16	13.84	38426.60	<0.001	0.13	0.11	0.15	12.88	38435.12	<0.001
Age * Charity * DTC	0.03	0.01	0.05	2.52	38426.60	0.01	0.04	0.02	0.06	3.40	38435.12	<0.001
Age * Charity * DRC	0.05	0.03	0.07	4.74	38426.60	<0.001	0.06	0.04	0.08	5.42	38435.12	<0.001

Note. CI low / up: 95% confidence interval lower / upper, F: female, M: male, DTW: death total worldwide, DTC: death total in country, DRC: death rate in country, CW: country wealth (gross national income per capita), I: international charity, N: national charity. The effect of death rate worldwide and any interactions including this term did not significantly improve the model fit.

COVID-19 severity and country-level wealth predict total donations and national bias in giving

In addition to the three-way interactions between age, charity location and COVID-19 deaths predicting donations reported in the main text, the model also showed significant two-way interactions and main effects (Table S6). Two-way interactions between COVID-19 severity and charity location showed that higher death totals, both in the participants' country ($\beta=0.06$ | 0.04, $ps<0.001$) and worldwide ($\beta=0.05$ | 0.06, $ps<0.001$), were associated with reduced national bias. Total deaths worldwide also showed a significant negative effect on overall donations ($\beta=-0.03$ | -0.04, $p=0.007$ | <0.001; Table S6). Participants in wealthier countries kept more money for themselves ($\beta=-0.18$ | -0.17, $ps<0.001$). Increased donations in less wealthy countries were predominantly towards the national charity, leading to greater national bias (interaction $\beta=0.14$ | 0.13, $ps<0.001$).

Most individual difference measures remain stable over time

For most of the individual differences measured, intraclass correlation coefficients (ICCs) revealed good reliability (ICC > 0.70). The eight measures with lower ICCs (0.34 – 0.67) were single-item measures, seven of which were the Morality as Cooperation items (see Table S7).

Table S7.
Results from test-retest reliability analysis (n=448)

Measure	ICC	Number of items
Collective narcissism	0.87	3
MaC – deference	0.50	1
MaC – fairness	0.34	1
MaC – family	0.37	1
MaC – group	0.42	1
MaC – heroism	0.39	1
MaC – property	0.47	1
MaC – reciprocity	0.44	1
Moral circle	0.67	1
Moral identity	0.85	10
Narcissism	0.84	6
National identity	0.92	2
Open-mindedness	0.76	6
Optimism	0.86	2
Political ideology	0.82	1
Self-control	0.88	4
Self-esteem	0.77	1
Social belonging	0.90	4
Wellbeing	0.88	2

Note. ICC: Intraclass correlation coefficient, MaC: Morality as Cooperation.

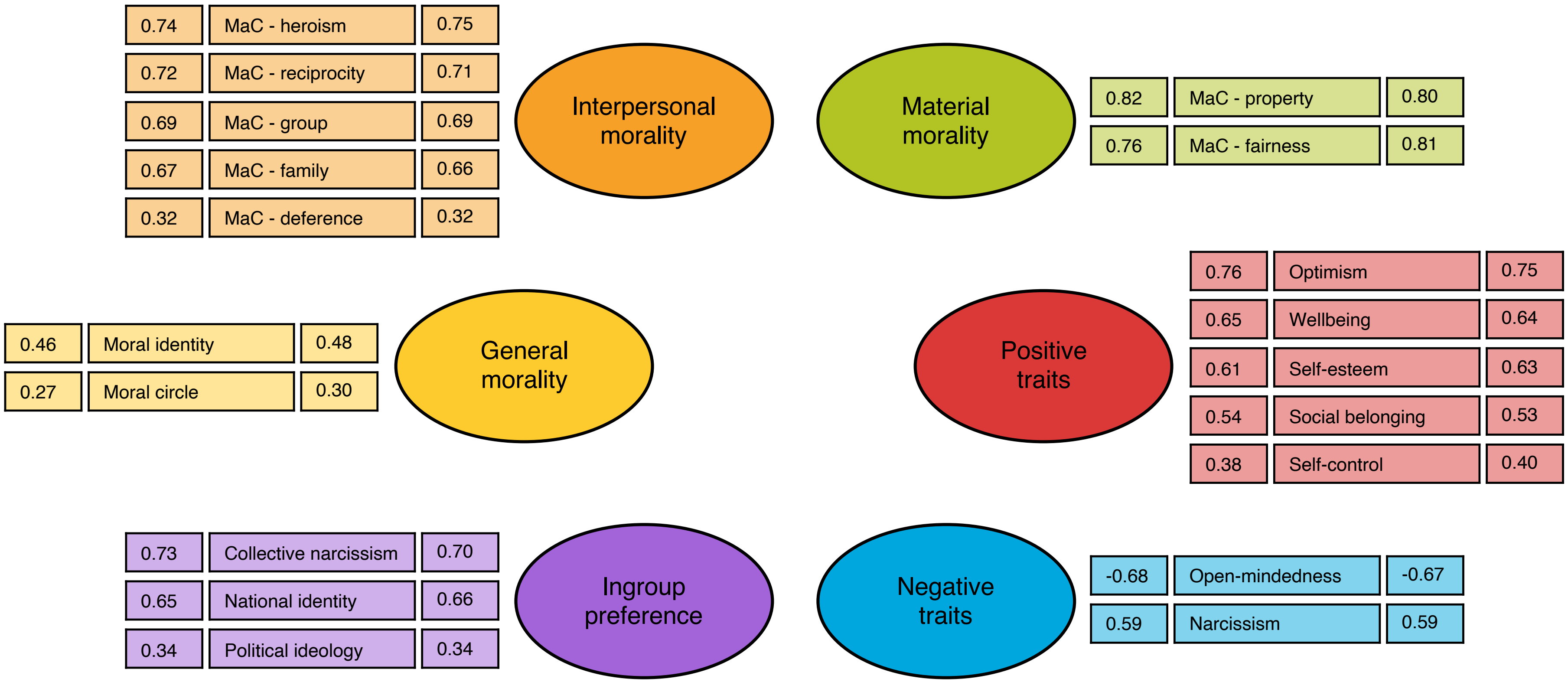

Fig. S5. Factor analysis of individual difference measures. Loadings of each measure are provided for subsample 1 on the left and subsample 2 on the right. Only loadings > 0.3 are reported, with the exception of moral circle in subsample 1 (see Methods). Note. MaC: Morality as Cooperation.

Individual difference measures correlate with age and prosocial behaviours

To test the relevance of the individual different factors for age-related changes in prosocial behaviour, we calculated correlations of the factor scores with age and the prosocial measures (Table S8 and see Results). We next tested whether there were differences in the strength of correlations between the prosocial measures for each factor. As this analysis was exploratory, we only report differences significant at $p<0.0001$ Bonferroni-corrected. For positive traits, negative traits, and interpersonal morality, these comparisons showed significant differences in the absolute size of the correlations such that distancing > national donations > international donations (Table S8). For example, positive traits showed a correlation of $r=0.20$ | 0.19 with distancing, $r=0.16$ | 0.15 with national donations, and $r=0.07$ | 0.08 with international donations. The pattern was similar for interpersonal morality. Negative traits showed a correlation of $r=-0.25$ | −0.24 with distancing, −0.13 | −0.14 with national donations, and −0.05 | −0.05 with international donations. General morality was more strongly related to distancing ($r=0.26$ | 0.27) but similarly related to both types of donations (national $r=0.15$ | 0.15; international $r=0.15$ | 0.15). In contrast, ingroup preference was positively associated with national donations ($r=0.11$ | 0.09) but as expected, negatively associated with international donations ($r=-0.12$ | −0.11), creating a significant difference between these two correlations.

Table S8.

Correlations of factor scores with age, distancing, donations to national charities, and donations to international charities

	Age		Distancing			Nat. donations			Intl. donations	
	S1	S2	S1	S2		S1	S2		S1	S2
Positive traits	0.11*	0.10*	0.20*	0.19*	†	0.16*	0.15*	‡	0.07*	0.08*
Negative traits	-0.13*	-0.11*	-0.25*	-0.24*	†	-0.13*	-0.14*	‡	-0.05*	-0.05*
Ingroup preference	0.04*	0.03*	0.08*	0.09*		0.11*	0.09*	‡	-0.12*	-0.11*
Interp. morality	0.06*	0.06*	0.23*	0.23*	†	0.13*	0.11*	‡	0.04*	0.05*
Material morality	-0.11*	-0.13*	0.04*	0.04*	†	0.00	-0.01		0.03*	0.03*
General morality	0.01	0.01	0.26*	0.27*	†	0.15*	0.15*		0.15*	0.15*

Note. Values are Pearson's correlation coefficients, Nat.: national, Intl.: international, S1: subsample 1, S2: subsample 2, Interp. morality: Interpersonal morality; * indicates significance at p<0.01 Bonferroni-corrected across all 24 correlations in each subsample, † indicates a significant difference between the correlations with distancing and national donations in both subsamples, ‡ indicates a significant difference between the correlations with national donations and international donations (comparisons between distancing and international donations not shown) p<0.0001 Bonferroni-corrected across 18 comparisons in each subsample.

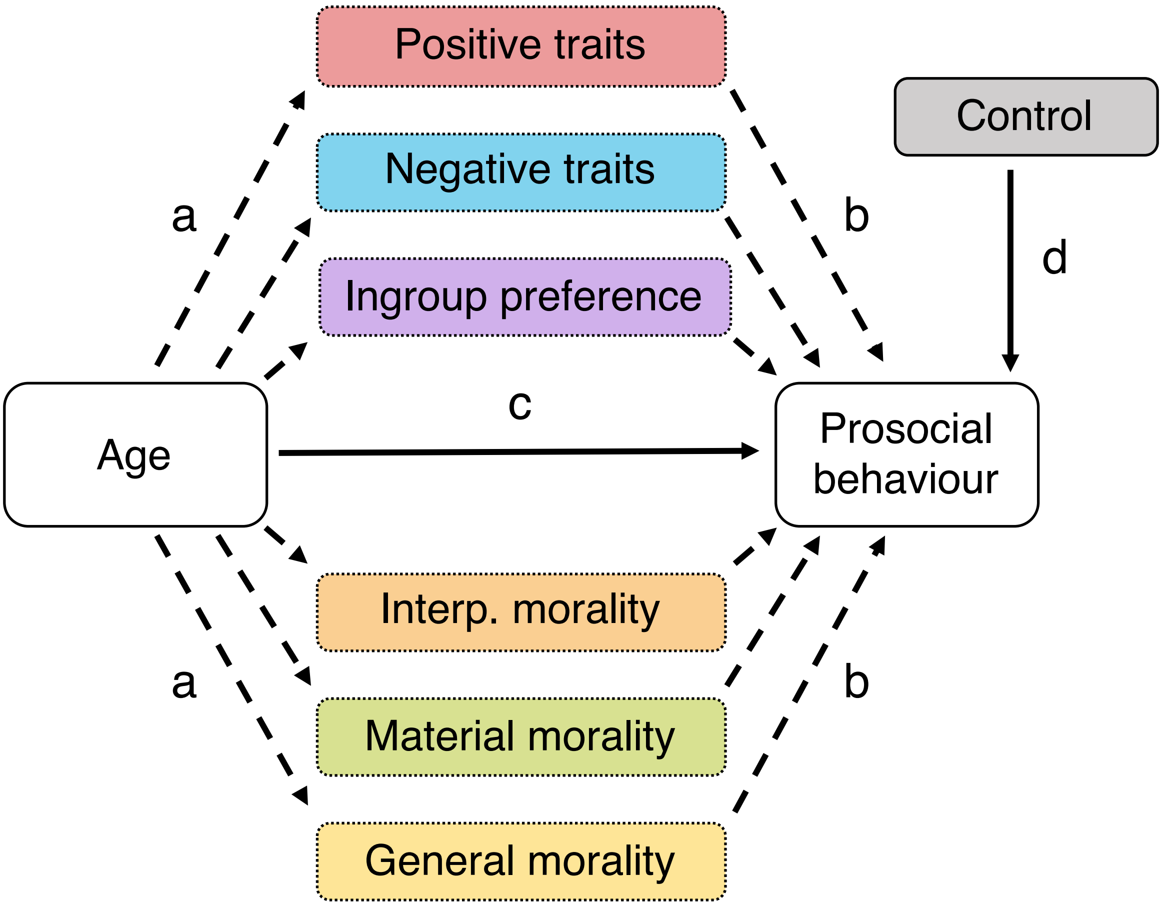

Fig. S6 Structural equation model of age effects on prosocial behaviour including trait factors and control variable. For each measure of prosocial behaviour (distancing, national donations, international donations) we included **a)** paths from age to the six trait factors, **b)** paths from the trait factors to the prosocial behaviour, **c)** a direct path from age to the prosocial behaviour, and **d)** a path from the control variable to prosocial behaviour. For distancing the control variable was perceived risk and for both national and international donations it was subjective wealth as in the main models. The indirect effects are a*b for each trait factor, the product of the dashed paths (see Table S9). Direct effects are shown with solid lines. Interp. morality: Interpersonal morality.

Table S9.
Paths coefficients from structural equation models

	Distancing		Nat. donations		Intl. donations	
**a) Age → factor**	S1	S2	S1	S2	S1	S2
Positive traits	0.11*	0.10*	0.11*	0.10*	0.11*	0.10*
Negative traits	-0.13*	-0.11*	-0.13*	-0.11*	-0.12*	-0.11*
Ingroup preference	0.04*	0.03*	0.04*	0.03*	0.04*	0.03*
Interpersonal morality	0.06*	0.06*	0.05*	0.05*	0.05*	0.05*
Material morality	-0.11*	-0.13*	-0.11*	-0.13*	-0.11*	-0.13*
General morality	0.01	0.01	0.01	0.01	0.01	0.01
**b) Factor → prosocial behaviour**						
Positive traits	0.03*	0.02	0.04*	0.02	0.05*	0.05*
Negative traits	-0.26*	-0.25*	-0.16*	-0.16*	0.06*	0.06*
Ingroup preference	0.11*	0.11*	0.15*	0.13*	-0.18*	-0.18*
Interpersonal morality	0.07*	0.08*	0.02	0.01	0.01	0.02
Material morality	0.06*	0.05*	0.01	0.00	0.00	0.00
General morality	0.14*	0.15*	0.07*	0.08*	0.19*	0.20*
**c) Age → prosocial behaviour**	0.04*	0.04*	0.03*	0.02*	-0.08*	-0.07*
**Total effect of interest (a * b + c)**	0.08*	0.07*	0.06*	0.05*	-0.09*	-0.07*
**d) Control → prosocial behaviour**	0.04*	0.03*	-0.05*	-0.06*	-0.04*	-0.04*

Note. Nat.: national, Intl.: international, S1: subsample 1, S2: subsample 2; values are standardised coefficients from structural equation models. A structural equation model was created for each prosocial behaviour – distancing, national donations, and international donations – separately. Each included **a)** paths from age to the trait factors, **b)** paths from the trait factor to the prosocial behaviour, **c)** a direct path from age to the prosocial behaviour, and **d)** a path from the control variable to prosocial behaviour (see Fig. S6). As in the main linear mixed-effects models, the control variable was perceived risk for distancing and subjective wealth for both national and international donations. We Bonferroni-corrected the significance values across all paths of that type from all three models in each subsample. For example, all 18 "b" paths, all six "c" paths. * indicates a significant path at p<0.01 corrected. We also applied a threshold of p<0.01 Bonferroni-corrected to the 18 indirect effects (a*b) in each subsample. Indirect paths (a & b) where the overall indirect effect (a*b) was not significant are in grey (for example general morality significantly predicted prosocial behaviour but general morality is not predicted by age so there is no indirect effect).

Table S10.

Results from linear mixed-effects models excluding participants who reported being a student

	**Subsample 1**						**Subsample 2**					
**Distancing**	beta	CI low	CI up	*t*	df	*p*	beta	CI low	CI up	*t*	df	*p*
Intercept	-0.1	-0.17	-0.03	-2.94	71.84	0.004	-0.08	-0.15	-0.01	-2.33	71.63	0.02
Gender (F > M)	0.28	0.25	0.30	20.13	19960.82	<0.001	0.28	0.25	0.30	20.14	19959.09	<0.001
Perceived risk	0.02	0.00	0.05	1.64	50.75	0.11	0.02	0.00	0.05	1.96	47.56	0.06
Age	0.10	0.07	0.14	6.29	55.61	<0.001	0.10	0.07	0.13	6.59	45.68	<0.001
**Total donations**												
Intercept	-0.08	-0.15	0.00	-1.99	74.12	0.05	-0.05	-0.13	0.02	-1.37	75.22	0.17
Gender (F > M)	0.14	0.11	0.16	9.94	19961.05	<0.001	0.14	0.12	0.17	10.63	20005.64	<0.001
Subjective wealth	-0.08	-0.10	-0.06	-7.70	40.12	<0.001	-0.09	-0.11	-0.06	-6.47	51.45	<0.001
Age (linear)	0.06	0.04	0.08	6.48	50.47	<0.001	0.06	0.04	0.07	6.12	47.21	<0.001
Age (quadratic)	0.05	0.03	0.06	6.60	5215.73	<0.001	0.03	0.02	0.05	4.72	5322.30	<0.001
**Donations by charity**												
Intercept	0.29	0.24	0.34	11.68	75.88	<0.001	0.29	0.24	0.35	11.15	74.15	<0.001
Gender (F > M)	0.10	0.08	0.12	10.67	40741.74	<0.001	0.11	0.10	0.13	12.41	41095.13	<0.001
Subjective wealth	-0.05	-0.07	-0.04	-8.42	47.10	<0.001	-0.05	-0.07	-0.04	-6.35	49.67	<0.001
Age	0.07	0.06	0.09	7.92	76.77	<0.001	0.07	0.05	0.09	7.10	66.89	<0.001
Charity (I > N)	-0.66	-0.67	-0.64	-72.72	41243.08	<0.001	-0.65	-0.67	-0.64	-72.60	41258.32	<0.001
Age * Charity	-0.12	-0.14	-0.11	-13.68	41243.59	<0.001	-0.11	-0.12	-0.09	-11.75	41258.94	<0.001

Note. CI low / up: 95% confidence interval lower / upper, F: female, M: male, I: international charity, N: national charity.

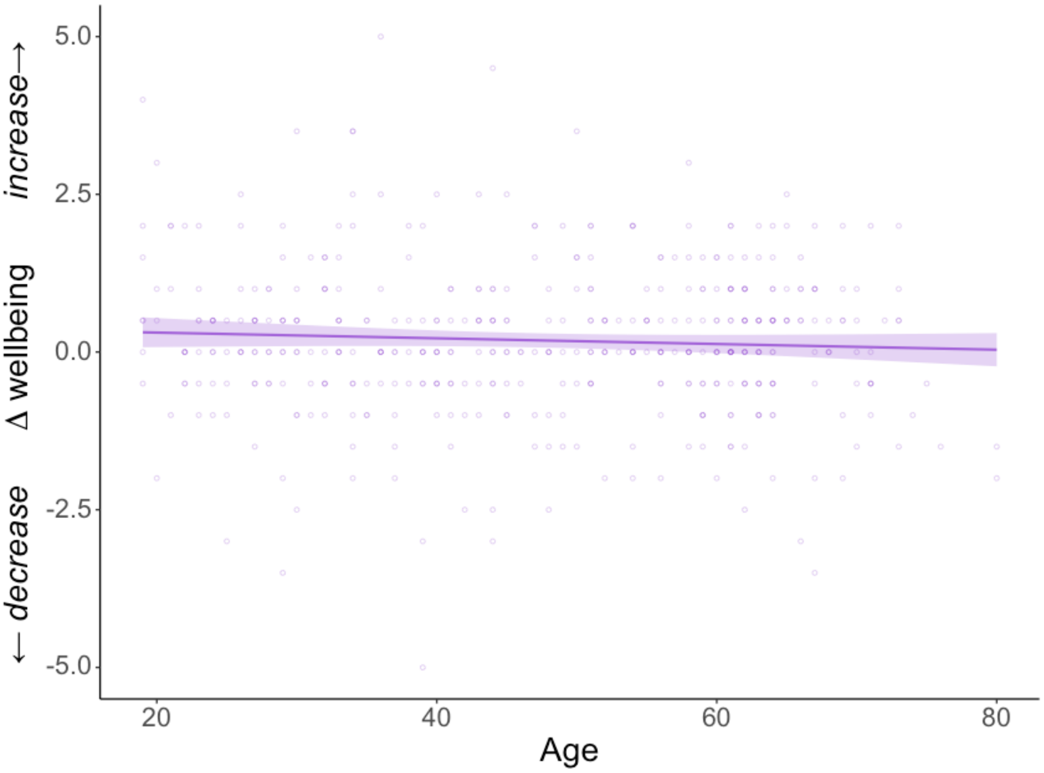

Fig. S7 Change in wellbeing over a month of the COVID-19 pandemic is not significantly associated with age. For the subset of participants who completed the survey at two time points, one month apart (n=448, UK only), we calculated a difference score for wellbeing - Δ wellbeing - by subtracting self-rated wellbeing at time 1 from their wellbeing at time 2. The Pearson's correlation between Δ wellbeing and age was not significant (r=-0.06, p=0.22) suggesting that any change in wellbeing over this time did not depend on age, so could not explain our main results. Line shows fitted linear model, shaded area shows 95% confidence interval.

Supplementary references

1. Schielzeth, H. *et al.* Robustness of linear mixed-effects models to violations of distributional assumptions. *Methods Ecol. Evol.* **11**, 1141–1152 (2020).
2. Harrison, F. & Mouden, C. E. Exploring the Effects of Working for Endowments on Behaviour in Standard Economic Games. *PLOS ONE* **6**, e27623 (2011).
3. Raihani, N. J., Mace, R. & Lamba, S. The Effect of $1, $5 and $10 Stakes in an Online Dictator Game. *PLOS ONE* **8**, e73131 (2013).
4. Maxwell, S. E. & Cole, D. A. Bias in cross-sectional analyses of longitudinal mediation. *Psychol. Methods* **12**, 23–44 (2007).
5. O'Laughlin, K. D., Martin, M. J. & Ferrer, E. Cross-Sectional Analysis of Longitudinal Mediation Processes. *Multivar. Behav. Res.* **53**, 375–402 (2018).
6. Lindenberger, U., von Oertzen, T., Ghisletta, P. & Hertzog, C. Cross-sectional age variance extraction: What's change got to do with it? *Psychol. Aging* **26**, 34–47 (2011).
7. Pek, J. & Hoyle, R. H. On the (In)Validity of Tests of Simple Mediation: Threats and Solutions: (In)Validity of Tests of Simple Mediation. *Soc. Personal. Psychol. Compass* **10**, 150–163 (2016).
8. Gamer, M., Lemon, J. & Singh, I. F. P. *irr: Various Coefficients of Interrater Reliability and Agreement.* (2019).
9. Weir, J. P. Quantifying test-retest reliability using the intraclass correlation coefficient and the SEM. *J Strength Con Res* 231–240 (2005).
