# Supplementary material for: Older adults across the globe exhibit increased prosocial behavior but also greater in-group preferences
Source: Nat Aging. 2021 Oct 11;1(10):880–8. doi: 10.1038/s43587-021-00118-3 (PMC10154238; doi:10.1038/s43587-021-00118-3)
Supplement: Supplementary file 2 — Reporting Summary [file 43587_2021_118_MOESM2_ESM.pdf]

nature research

Corresponding author(s): Dr Jo Cutler & Dr Patricia Lockwood

Last updated by author(s): Aug 4, 2021

Reporting Summary

Nature Research wishes to improve the reproducibility of the work that we publish. This form provides structure for consistency and transparency in reporting. For further information on Nature Research policies, see our Editorial Policies and the Editorial Policy Checklist.

Statistics

For all statistical analyses, confirm that the following items are present in the figure legend, table legend, main text, or Methods section.

n/a	Confirmed	
☐	☒	The exact sample size (*n*) for each experimental group/condition, given as a discrete number and unit of measurement
☐	☒	A statement on whether measurements were taken from distinct samples or whether the same sample was measured repeatedly
☐	☒	The statistical test(s) used AND whether they are one- or two-sided<br>*Only common tests should be described solely by name; describe more complex techniques in the Methods section.*
☐	☒	A description of all covariates tested
☐	☒	A description of any assumptions or corrections, such as tests of normality and adjustment for multiple comparisons
☐	☒	A full description of the statistical parameters including central tendency (e.g. means) or other basic estimates (e.g. regression coefficient) AND variation (e.g. standard deviation) or associated estimates of uncertainty (e.g. confidence intervals)
☐	☒	For null hypothesis testing, the test statistic (e.g. *F*, *t*, *r*) with confidence intervals, effect sizes, degrees of freedom and *P* value noted<br>*Give P values as exact values whenever suitable.*
☒	☐	For Bayesian analysis, information on the choice of priors and Markov chain Monte Carlo settings
☐	☒	For hierarchical and complex designs, identification of the appropriate level for tests and full reporting of outcomes
☐	☒	Estimates of effect sizes (e.g. Cohen's *d*, Pearson's *r*), indicating how they were calculated

Our web collection on statistics for biologists contains articles on many of the points above.

Software and code

Policy information about availability of computer code

Data collection	Participants completed a survey online, with many countries using Qualtrics (Provo, Utah; April 2020) or a similar platform
Data analysis	Data prepreparation was conducted in MATLAB 2019b using custom scripts. Data analysis was conducted in R (v3.6.2) with R studio (v1.4.1106; packages ggplot2, lme4, parameters, effectsize, psych, lavaan). All scripts can be found on the Open Science Framework & Github https://doi.org/10.17605/OSF.IO/9WVP4

For manuscripts utilizing custom algorithms or software that are central to the research but not yet described in published literature, software must be made available to editors and reviewers. We strongly encourage code deposition in a community repository (e.g. GitHub). See the Nature Research guidelines for submitting code & software for further information.

Data

Policy information about availability of data

All manuscripts must include a data availability statement. This statement should provide the following information, where applicable:

- Accession codes, unique identifiers, or web links for publicly available datasets
- A list of figures that have associated raw data
- A description of any restrictions on data availability

The survey data is available from the International Collaboration on Social & Moral Psychology: COVID-19 website: https://icsmp-covid19.netlify.app/. Data on COVID-19 rates are available from the Global Change Data Lab Our World in Data website: https://ourworldindata.org/coronavirus. Life expectancy and gross national income data are available from Worldometer: https://www.worldometers.info/demographics/life-expectancy/ and the World Bank: https://databank.worldbank.org/source/world-development-indicators respectively. The full, specific dataset used in this study and the preregistration are available from: https://doi.org/10.17605/OSF.IO/9WVP4. Source data are provided with this paper.

Field-specific reporting

Please select the one below that is the best fit for your research. If you are not sure, read the appropriate sections before making your selection.

☐ Life sciences ☒ Behavioural & social sciences ☐ Ecological, evolutionary & environmental sciences

For a reference copy of the document with all sections, see nature.com/documents/nr-reporting-summary-flat.pdf

Behavioural & social sciences study design

All studies must disclose on these points even when the disclosure is negative.

Study description	Quantitative survey including demographics and age, self-report measures of behaviour, measures of traits, and a charitable giving measure equivalent to a dictator game manipulating the recipient of donations (national and international charity).
Research sample	46,576 participants (age 18-99, 52% women, 48% men, <1% other genders) collected as representative samples in 67 countries. The sample was determined by the aim to recruit a representative sample in terms of age and gender and do so in as many countries as possible with countries determined by where researchers in the Collaboration had access to data collection.
Sampling strategy	The sample size was determined by the number of countries in which researcher teams volunteered to collect data with a target sample size of at least 500 participants in each country. Samples aimed to be representative of the population on age and gender and this was possible in 30 countries. Most countries used a recruitment platform, for example Prolific, to recruit and pay participants or other stratified sampling technique to generate a representative sample.
Data collection	All participants completed the study online and no researcher was present.
Timing	22nd April - 30th May 2020
Data exclusions	Data were excluded for participants who a) did not answer at least 75% of the survey, b) did not report their age, were aged under 18 or over 100, or c) failed an attention check. These criteria were established in advance. After exclusions there was a final sample of 46,576 from a total of 50,944 participants who completed the study.
Non-participation	No participants declined participation or withdrew from the compiled dataset.
Randomization	No randomization was necessary as the variable of interest was age.

Reporting for specific materials, systems and methods

We require information from authors about some types of materials, experimental systems and methods used in many studies. Here, indicate whether each material, system or method listed is relevant to your study. If you are not sure if a list item applies to your research, read the appropriate section before selecting a response.

Materials & experimental systems

n/a	Involved in the study	
☒	☐	Antibodies
☒	☐	Eukaryotic cell lines
☒	☐	Palaeontology and archaeology
☒	☐	Animals and other organisms
☐	☒	Human research participants
☒	☐	Clinical data
☒	☐	Dual use research of concern

Methods

n/a	Involved in the study	
☒	☐	ChIP-seq
☒	☐	Flow cytometry
☒	☐	MRI-based neuroimaging

Human research participants

Policy information about studies involving human research participants

Population characteristics	See above.
Recruitment	Samples in each country were recruited to be nationally representative in terms of age and gender.
Ethics oversight	University of Kent

Note that full information on the approval of the study protocol must also be provided in the manuscript.
